# Data for the potential gold mineralization mapping with the applications of Electrical Resistivity Imaging and Induced Polarization geophysical surveys

**DOI:** 10.1016/j.dib.2018.12.086

**Published:** 2018-12-31

**Authors:** Mohd Hariri Arifin, John Stephen Kayode, Muhammad Khairel Izwan, Hussein Ahmed Hasan Zaid, Hamzah Hussin

**Affiliations:** a School of Environmental Science and Natural Resources, Department of Geology, Universiti Kebangsaan Malaysia, Bangi, Selengor, Malaysia; b Environmental Technology, School of Industrial Technology, Universiti Sains Malaysia, 11800 Pulau-Pinang, Malaysia; c Geo Technology Resources sdn Bhd, 31-1, Jalan mawar 5B, Taman Mawar, 43900 Sepang, Selangor, Malaysia; d Program Geosains, Jabatan Geosains, Fakulti Sains Bumi, Universiti Malaysia Kelantan, 17600 Jeli, Kelantan, Malaysia

## Abstract

To identify the potential zones for gold mineralization at the Felda Chiku 3, Gua Musang, Kelantan, East coast Malaysia, twenty-one (21) geophysical survey lines were conducted at the proposed mineral exploration site using the pole - dipole of the electrical resistivity and induced polarization arrays to get the maximum depth of 150 m with 400 m survey length. From the resistivity and chargeability concentration maps, the potential mineralized zones as delineated, was observed to be dominantly concentrated towards the southwest and northern part of the area. The 3D resistivity and chargeability slice model present low resistivity values and high chargeability values that are well correlated which is palpable especially at the depths of 25 m and 50 m respectively. The data showed that the potential mineralized zones are trending approximately north-south directions. Forty (40) drilling locations were proposed for follow-up drilling based on the resistivity and chargeability models.

**Specifications table**

**Table:** 

**Subject area**	*Earth and Planetary Science*
**More specific subject area**	*Geophysics*
**Type of data**	*Table, text file and figures*
**How data was acquired**	*The 2D ER and IP data were acquired using Land imaging system by Survey with ABEM Terrameter SAS4000*
**Data format**	*Raw, filtered, analyzed*
**Experimental factors**	*The 2D ER and IP data were originally processed using RES2DINV software model and ArcGIS Software*
**Experimental features**	*Very brief experimental description*
	The 2D resistivity and chargeability slice model obtained was able to delineates the potential mineralized zones as presented in this data article, that was observed to be dominantly concentrated towards the southwest and the northern part of the area.
**Data source location**	*Felda Chiku 3, Gua Musang, Kelantan Darul Naim, East Coast Malaysia. The project area is located about 10km from Kampung Paloh 2 while 40km from Gua Musang town. Project area can be access by using Kota Bharu - Gua Musang Highways. Lat N 553266 and Long E 460884.*
**Data accessibility**	*The data is with this article.*
**Related Articles**	[1] Mohd Hariri Arifin, John Stephen Kayode, and Nawawi, N.M.N Gold Prospectivity Mapping with the Applications of Integrated 2D Electrical Resistivity; Induced Polarization, and the 3D GIS methods of approach to Exploration targets in the Peninsula Malaysian Gold Belts. (Article in final form, yet to be submitted to any journal)
	[2] S.W. Goh., G.H. Teh, W.F.W. Hassan. Gold Mineralization and Zonation in the State of Kelantan*. Bulletin of Geological Society of Malaysia***52**. (2006) 129-135.
	[3] K.S. Ariffin. Mesothermal Lode Gold Deposit Central Belt Peninsular Malaysia. In *Earth Science*. 2012 InTech. 313-342.DOI: 10.5772/26179.

**Value of the data**

• The choice of the geophysical techniques with the addition of 3-D GIS model to explored for the gold mineral is to improve prospectivity, the quality of the data and the depth of investigations.
• The data help in smart decisions and prioritizing the specific location to be drill for gold exploration.
• The data is extremely applicable in mineral exploration planning and policy formulations, mining site assessment and sensible gold mineralization project that could be implemented in any part of the World.
• The data is crucial in pre-gold-mining decisions that help to safe cost and time for the pinpointing the zones of mineral occurrence.

## 1 Data

**Fig. 1:**
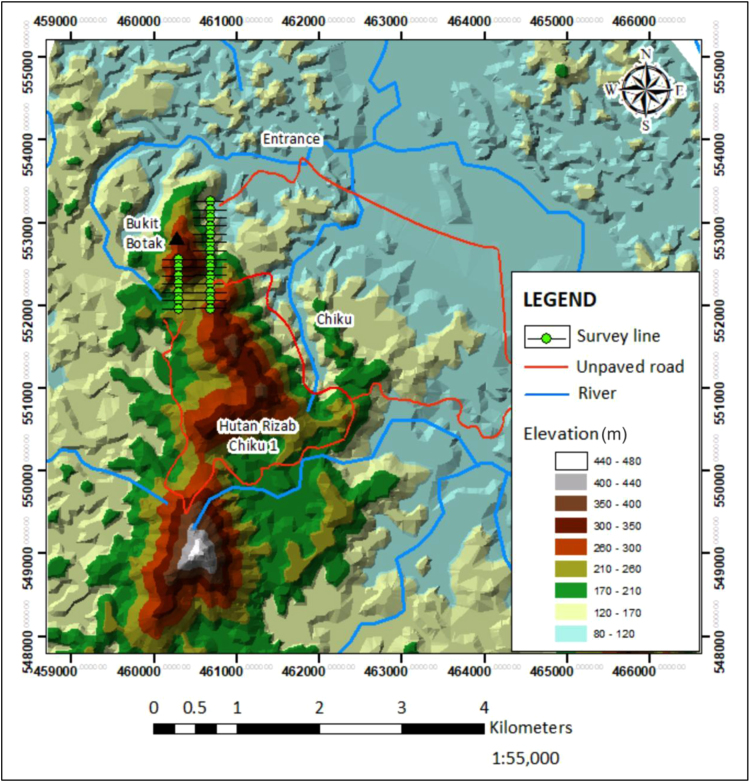
Topography map of the area showing the data collection locations.

**Fig. 2:**
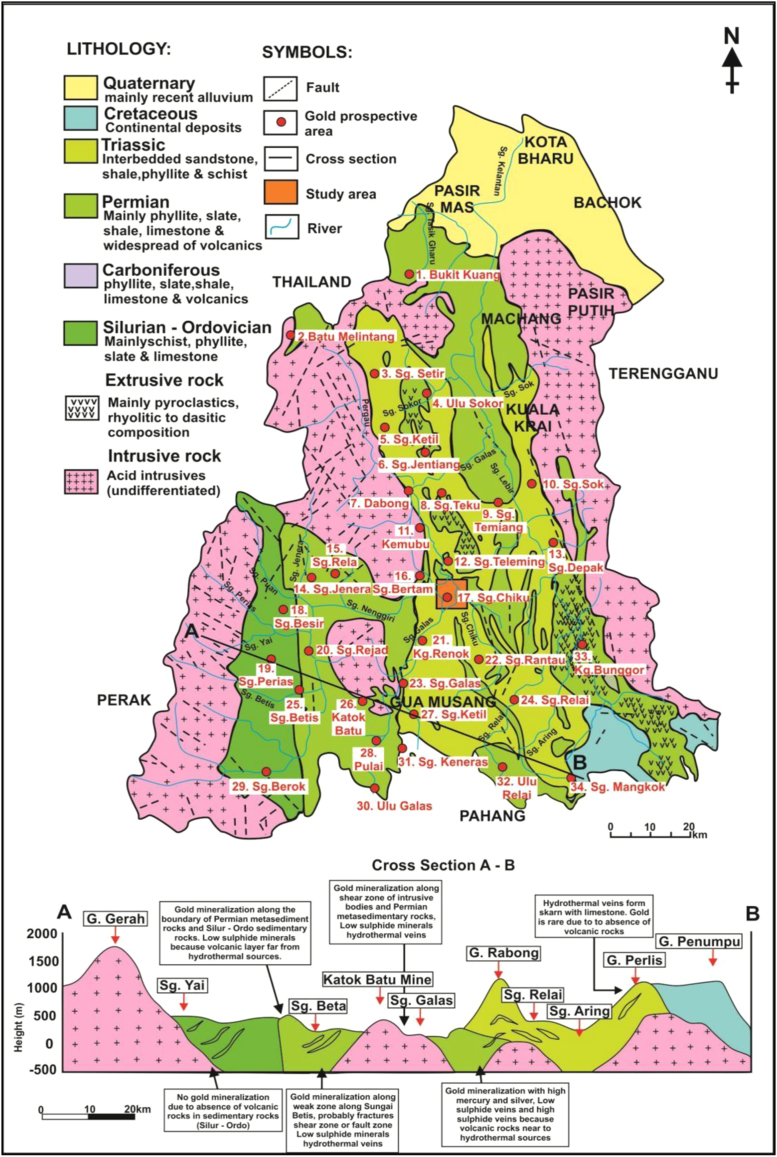
Geological map of Kelantan with distributions of the gold mineralization zones modified after Goh et al. [2] and Ariffin [3].

2-D Electrical Resistivity Imaging (E.R.I) and Induced Polarization (I.P) geophysical survey were conducted at Felda Chiku 3, Gua Musang, Kelantan, to identify the potential gold mineralization zones. Twenty-one (21) survey lines were conducted at the proposed site using pole - dipole array to get maximum depth of about 150 m with 400 m survey length lines. The survey was carried out using 41 and 61 electrodes respectively for the data collection on a multi-core cables. A resistivity meter system with internal microprocessor (ABEM Terrameter SAS4000) controlled the circuitry together with an electronic selector switching unit (ABEM LUND ES 10 – 64C) are used to automatically select the appropriate four electrodes for each measurement [1]. The subsurface geological structures of the area where the data was collected is principally in the state of Kelantan and are mainly distributed in the southern part of the central area as shown in Fig. 1, Fig. 2.

## 2 Experimental design, materials and methods

**Fig. 3:**
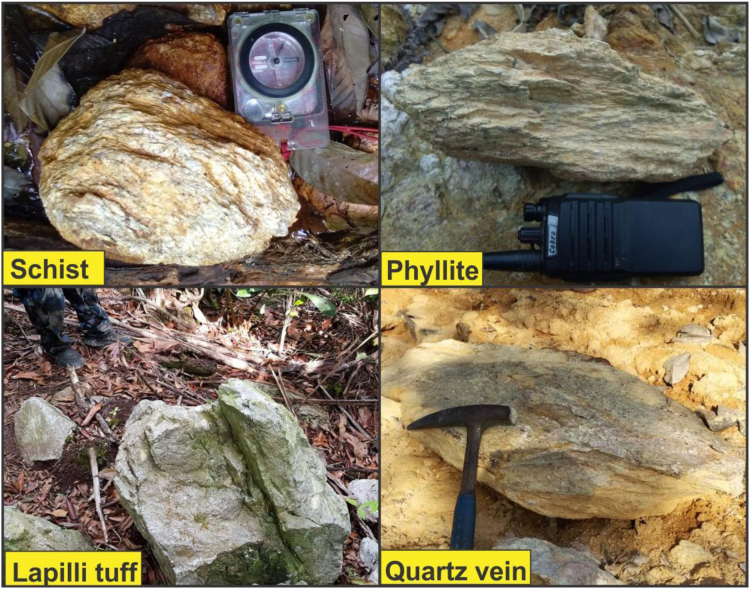
Several types of rocks exposed within the project area as observed during the geological mapping.

The location of the data collection area is in the southern part of central Kelantan State that covered the catchment area of Sungai Galas from the confluence of Sungai Chiku to upstream Sungai Galas. The gold deposits sited in the project area are principally within the hydrothermal veins that includes low sulphide and high sulphide quartz veins together with quartz veins in sheared granitoid and structurally controlled quartz veins. Basically, the sulphide comprises of modest and confined mineral contents within the veins. The high mercury and silver contents in this region could be perhaps due to basic intrusions that could possibly be situated at a depth of about 700 m below the ground surface. The lithologies of this area as showed in the field observations, comprises of lapilli tuff, phyllite, and schist (Fig. 3)**.** Furthermore, the majority of the phyllite and schist rocks units underlain the subsurface structural formations of the study area were occupied with quartz veins from the geological mapping. On the other hand, the quartz veins were filled-up along the foliation, cracks and fractures of the schist and the phyllite geological formations.

The field data collected were initially processed using RES2DINV software, model of inversion sections (pseudo section), with the distance and the depth generated, together with the topographic correction. The electrical resistivity data helped to delineates the geology and subsurface structures underlain the area, whereas the induced polarization data was used for defining the distributions of potential mineralized zones.

**Fig. 4:**
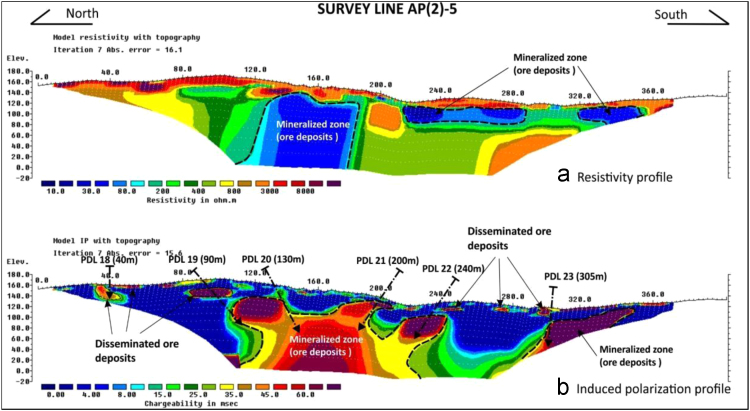
A typical of RES2DINV resistivity and Induced Polarization image for the gold exploration at Air Piau, Tanah Merah, Kelantan.

Generally, all the resistivity and chargeability data are as presented in the supplementary file in appendix II, with low resistivity and high chargeability anomalies. The dark red to purple colour on the RES2DINV image presented in Fig. 4, indicates zones with high resistivity data values > 2000 Ω-m and high chargeability data values > 30 ms. For the meantime, the dark blue to light blue colour showed areas with low resistivity data values < 200 Ω-m and low chargeability data values < 3 ms.
