# Supplementary figures and images for: Data for the potential gold mineralization mapping with the applications of Electrical Resistivity Imaging and Induced Polarization geophysical surveys
Source: Data Brief. 2018 Dec 31;22:830–5. doi: 10.1016/j.dib.2018.12.086 (PMC6362863; doi:10.1016/j.dib.2018.12.086)

# **Supplementary Resistivity and Induced Polarization**


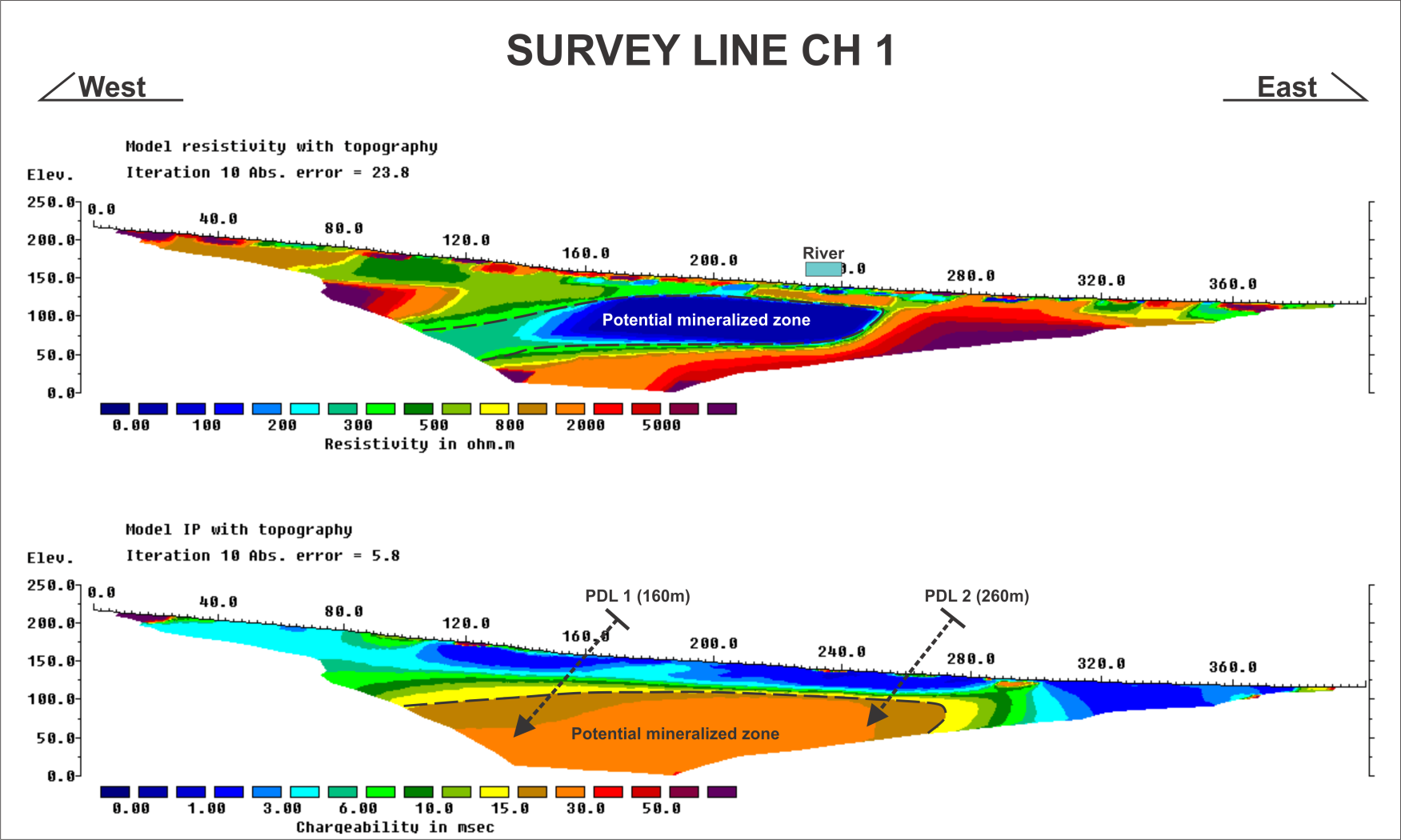

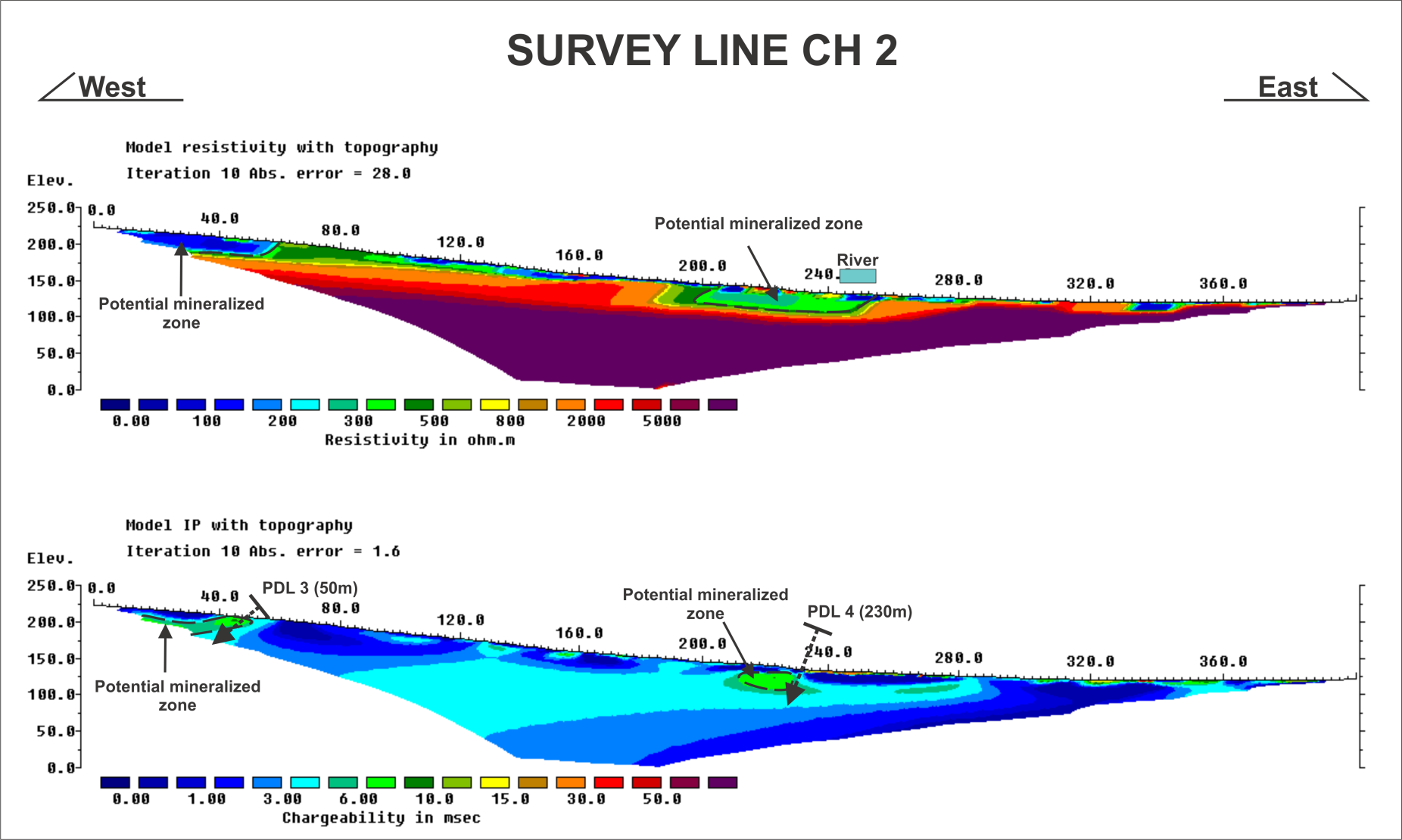

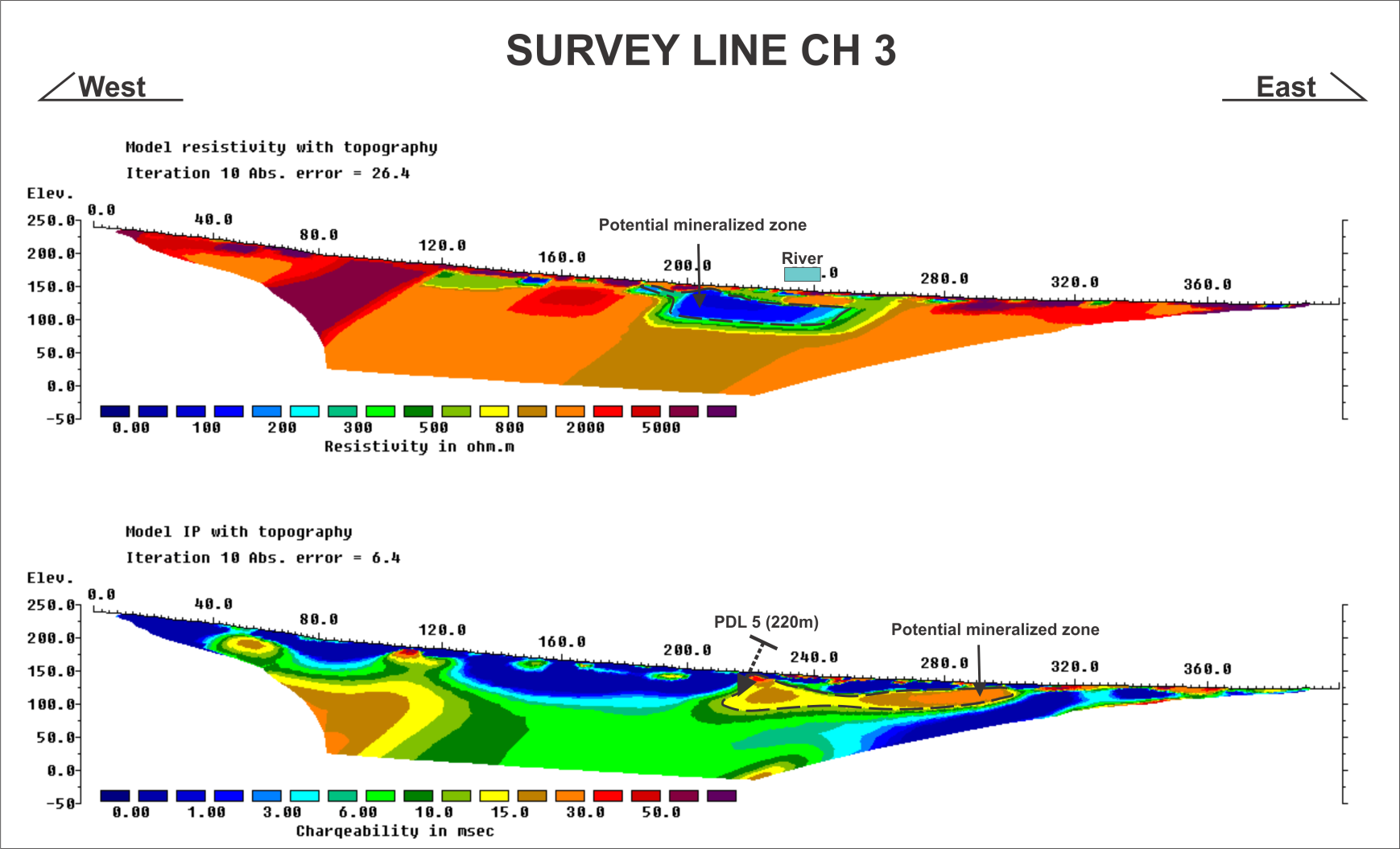

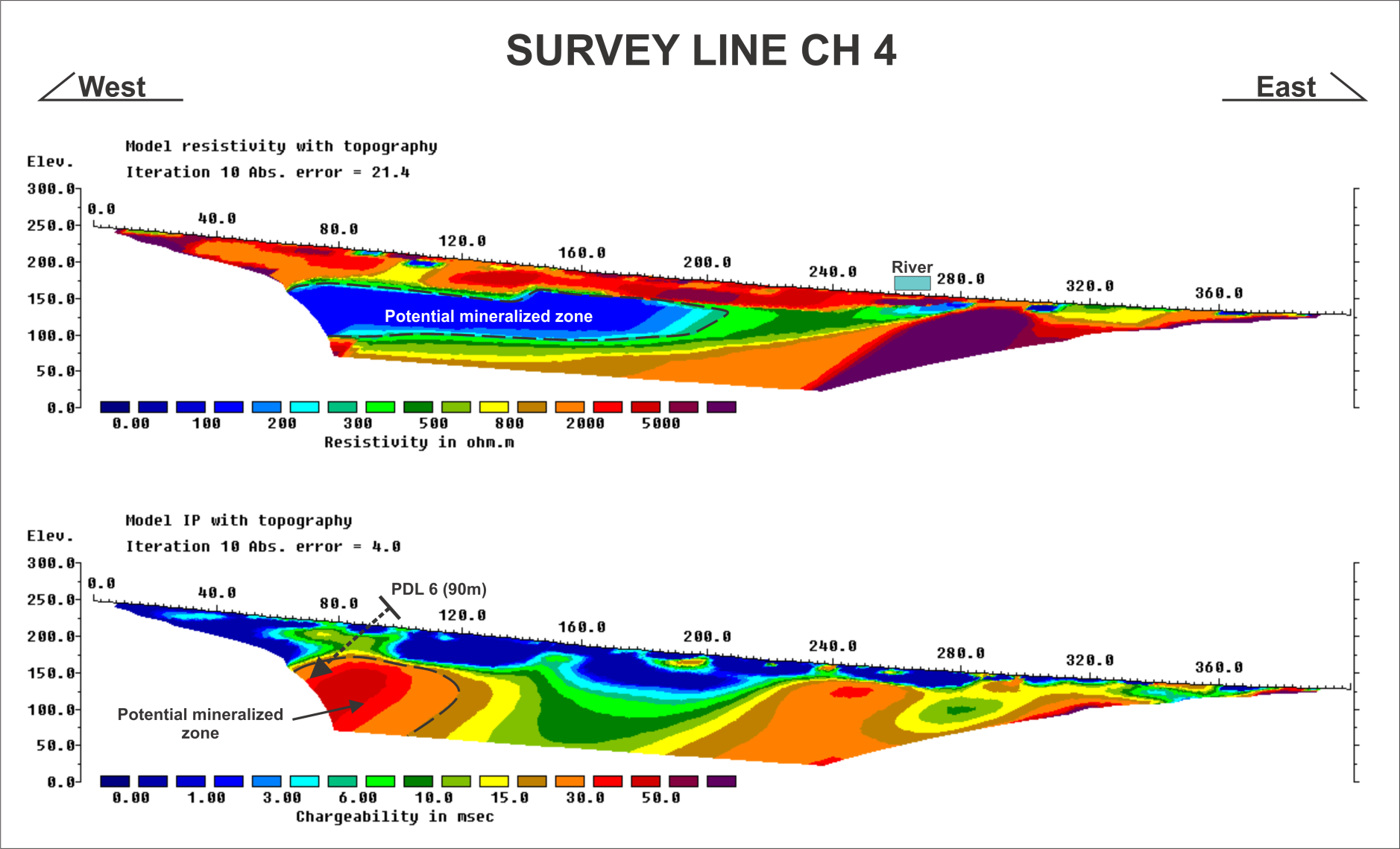

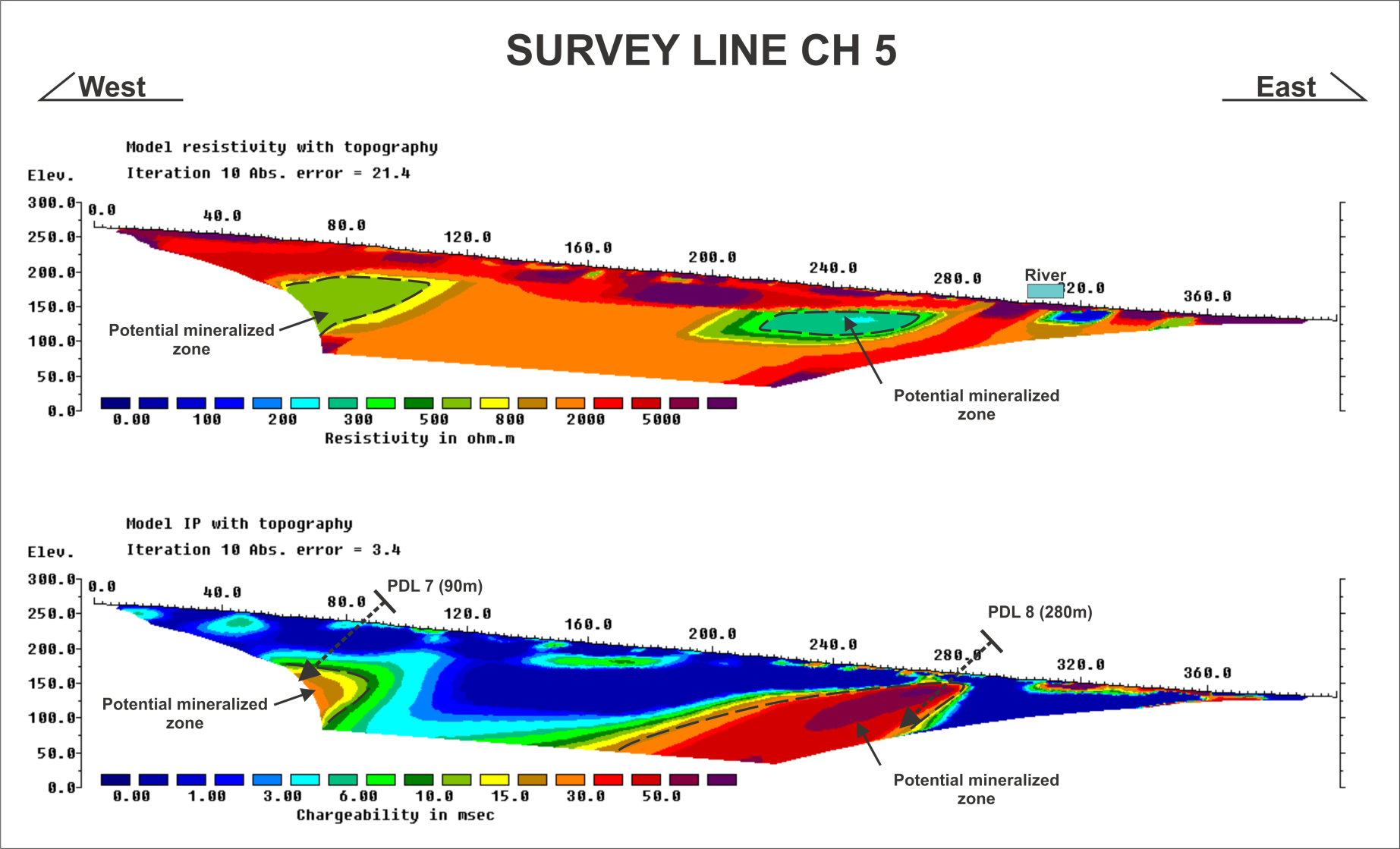

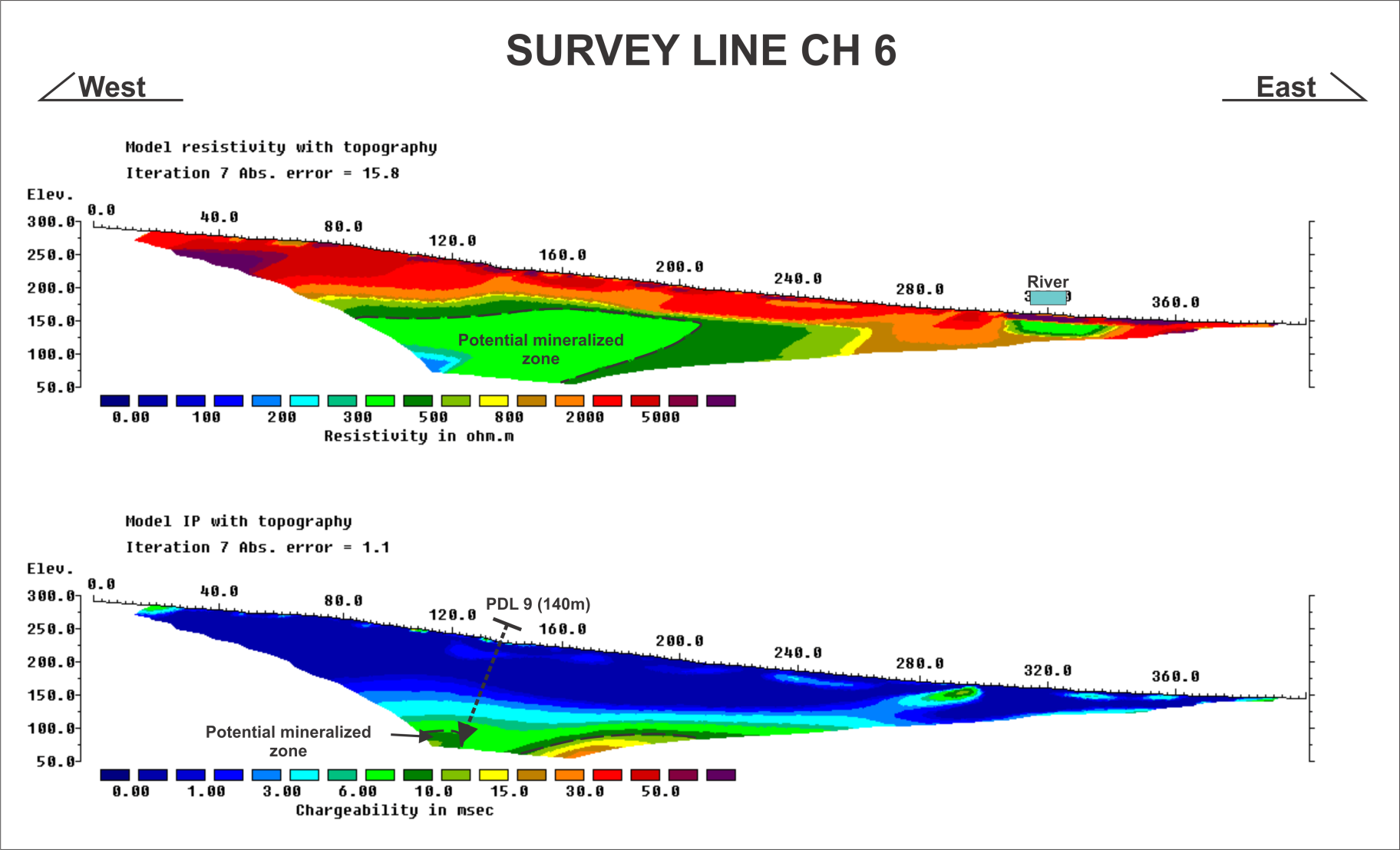

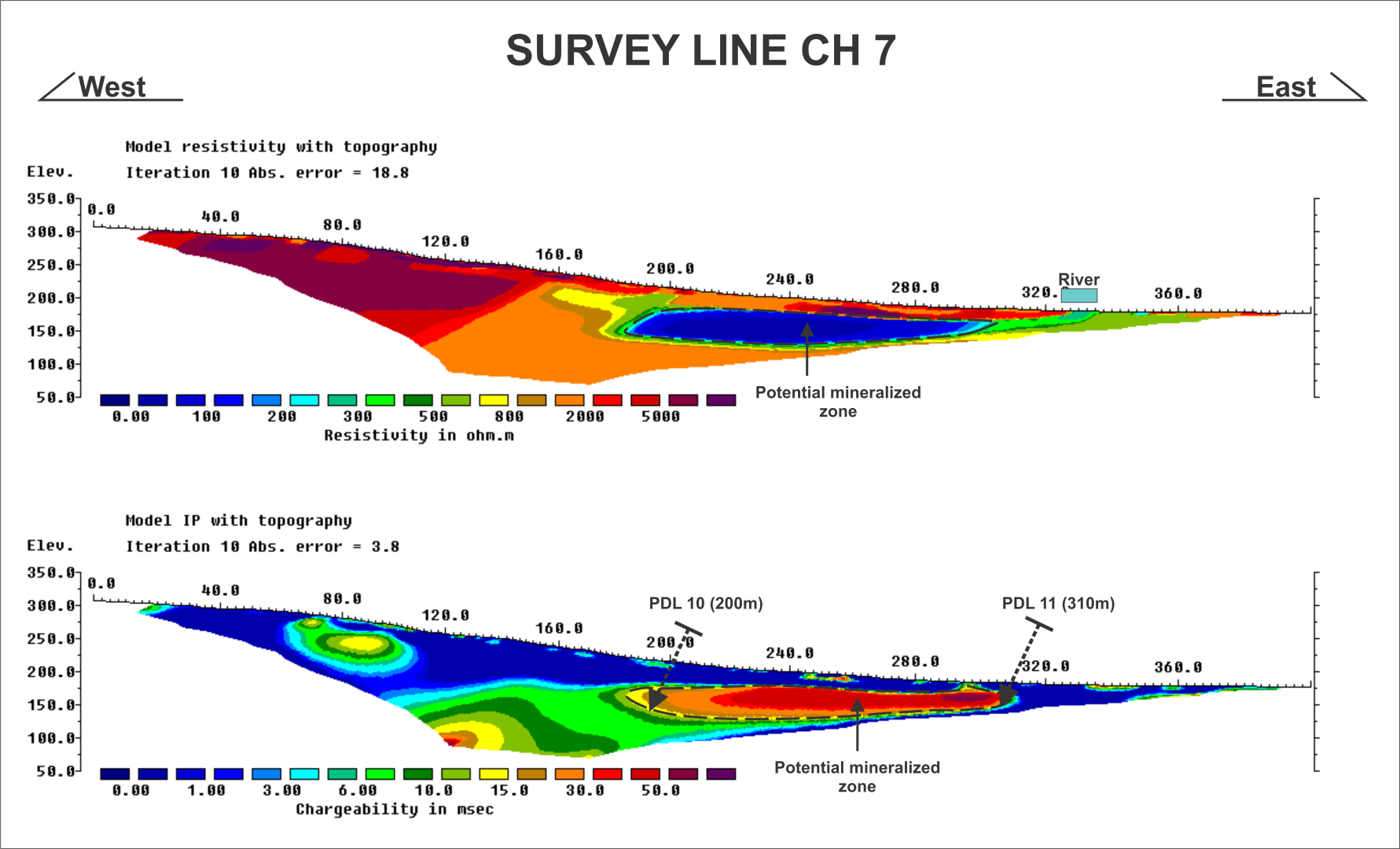

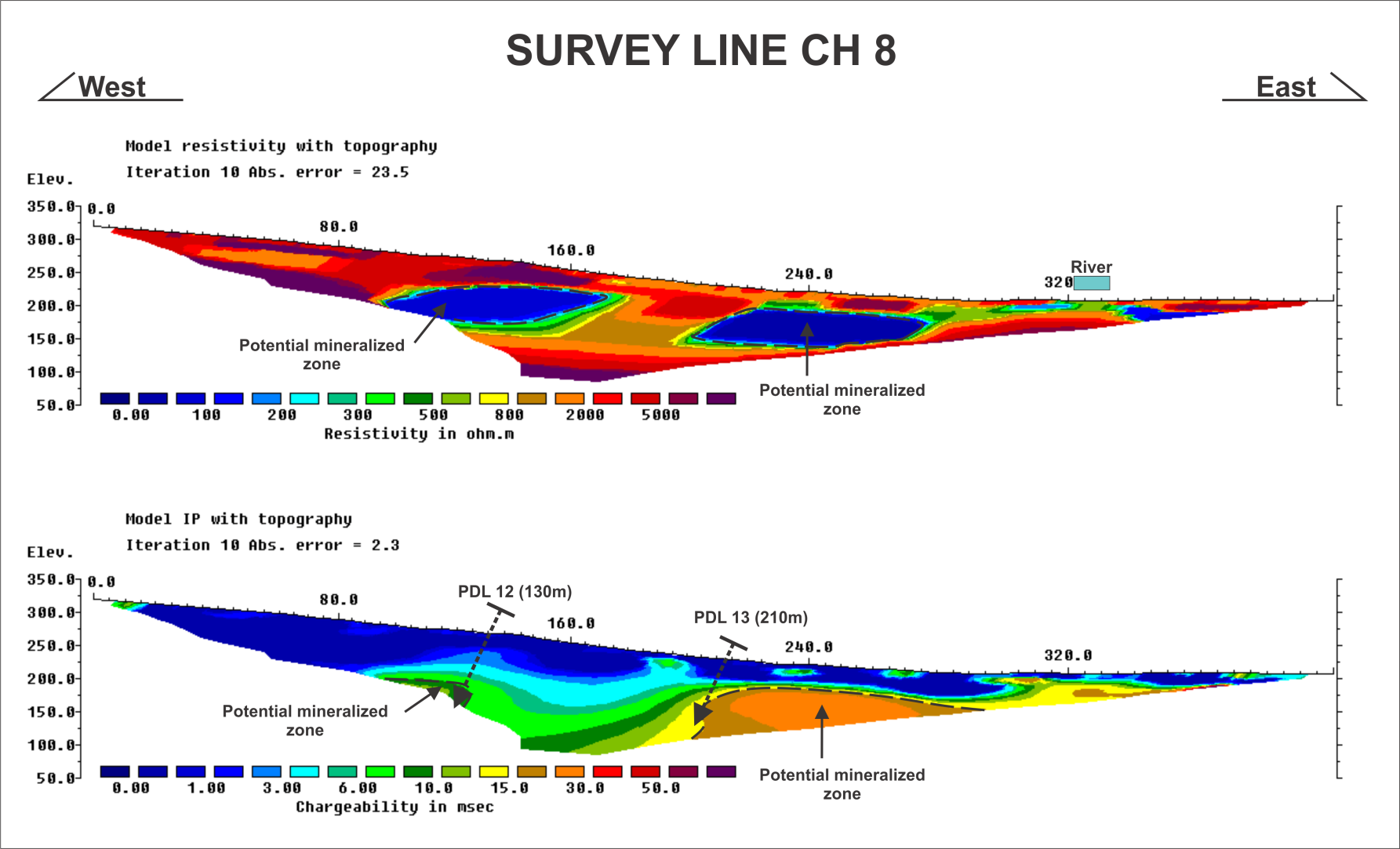

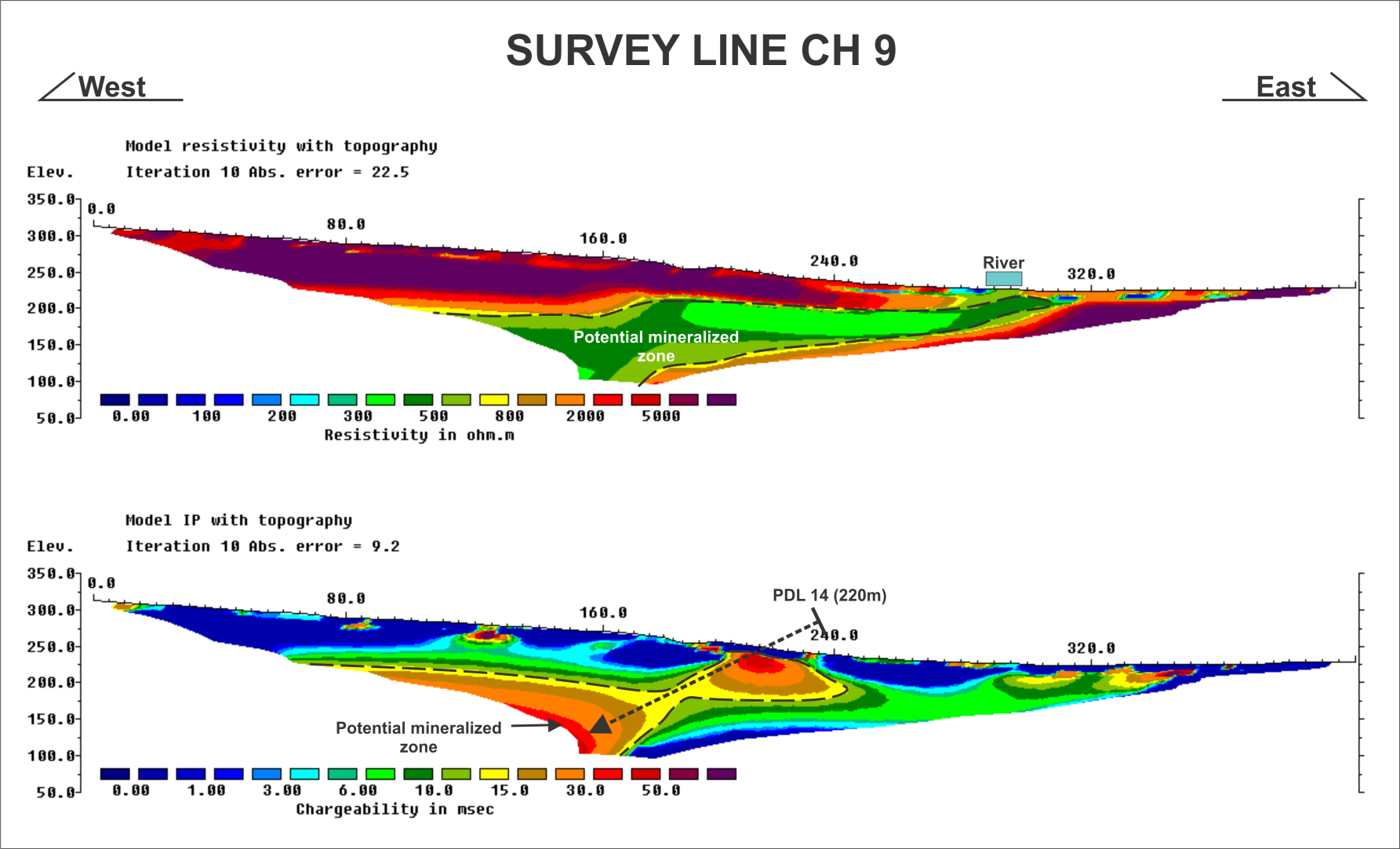

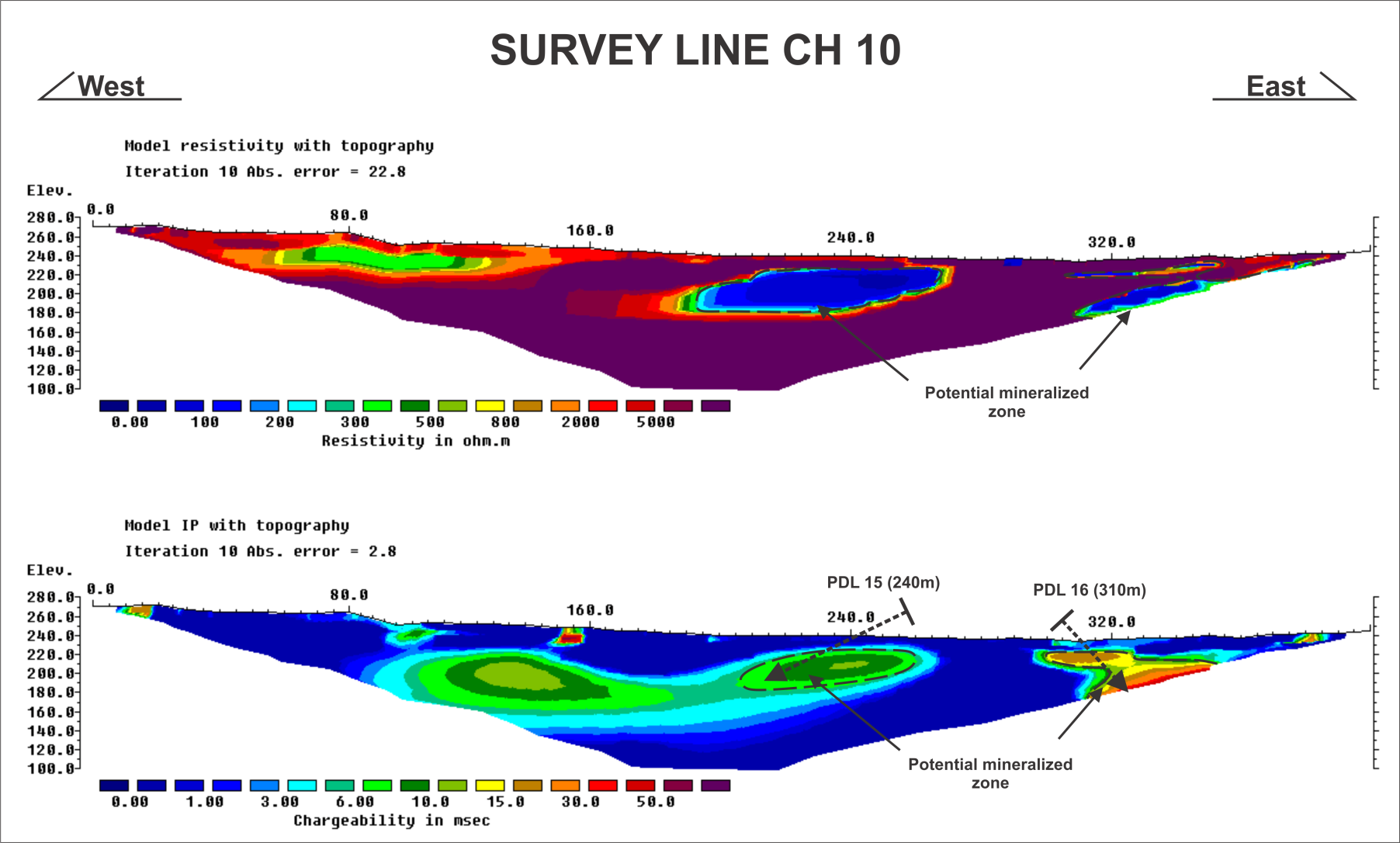

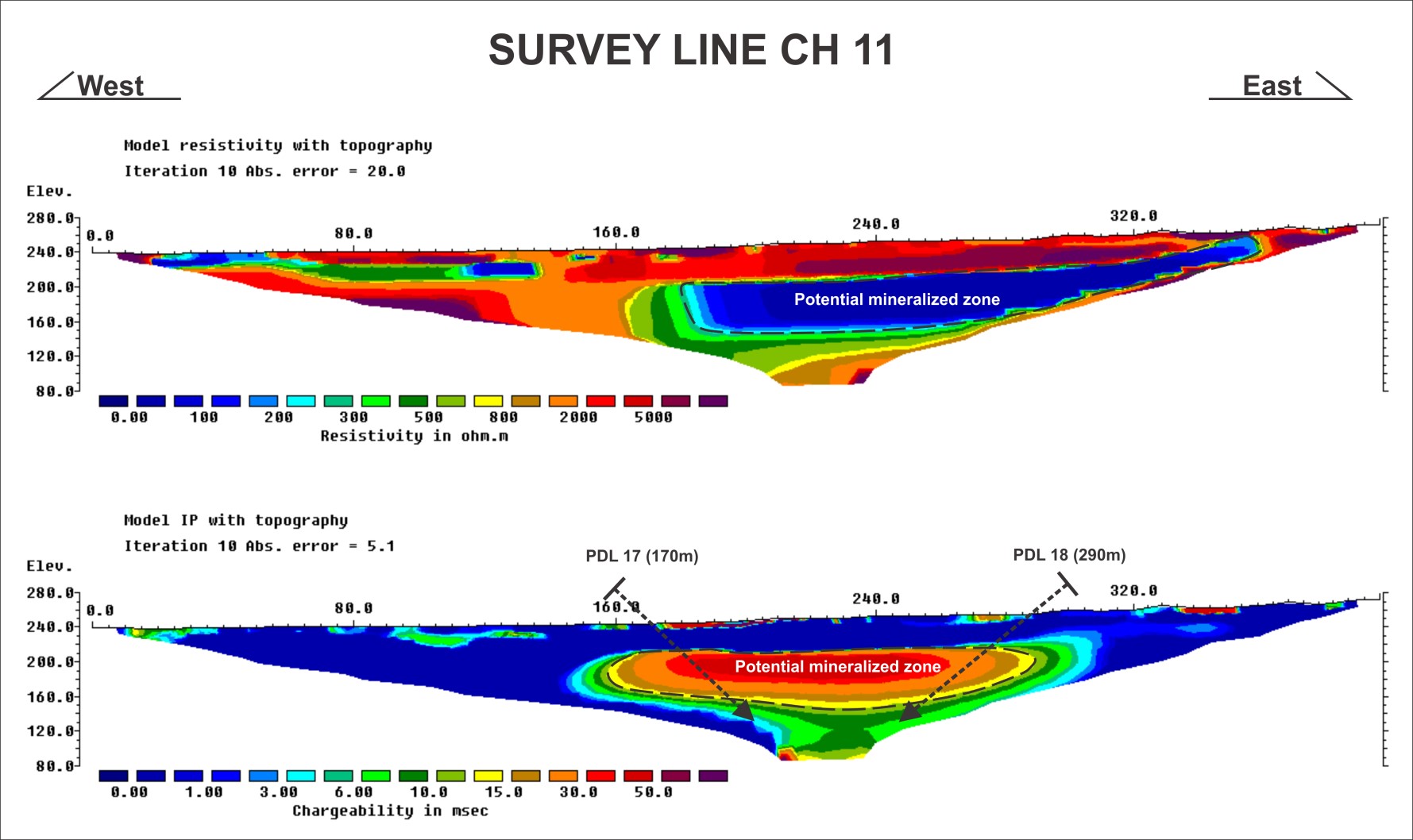

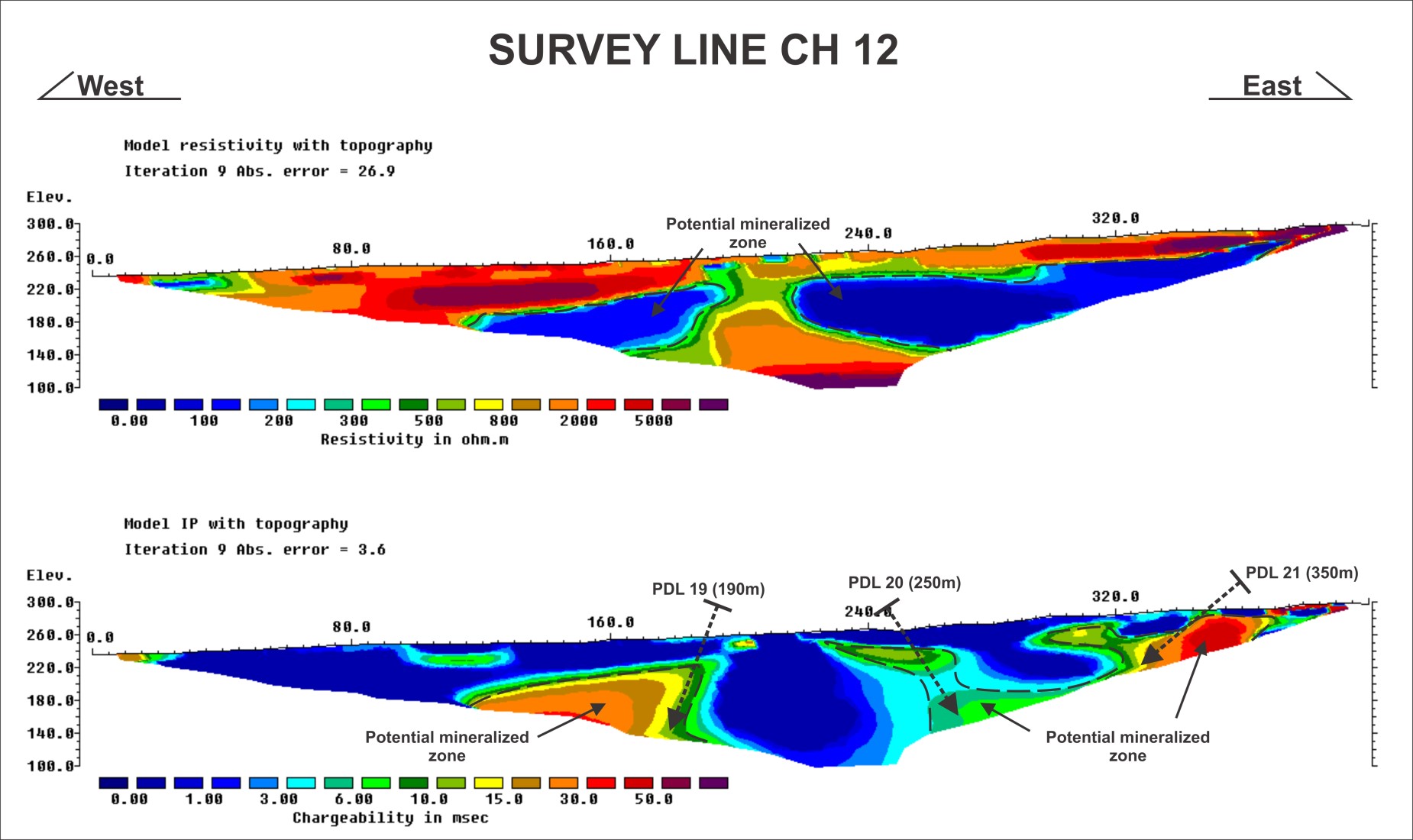

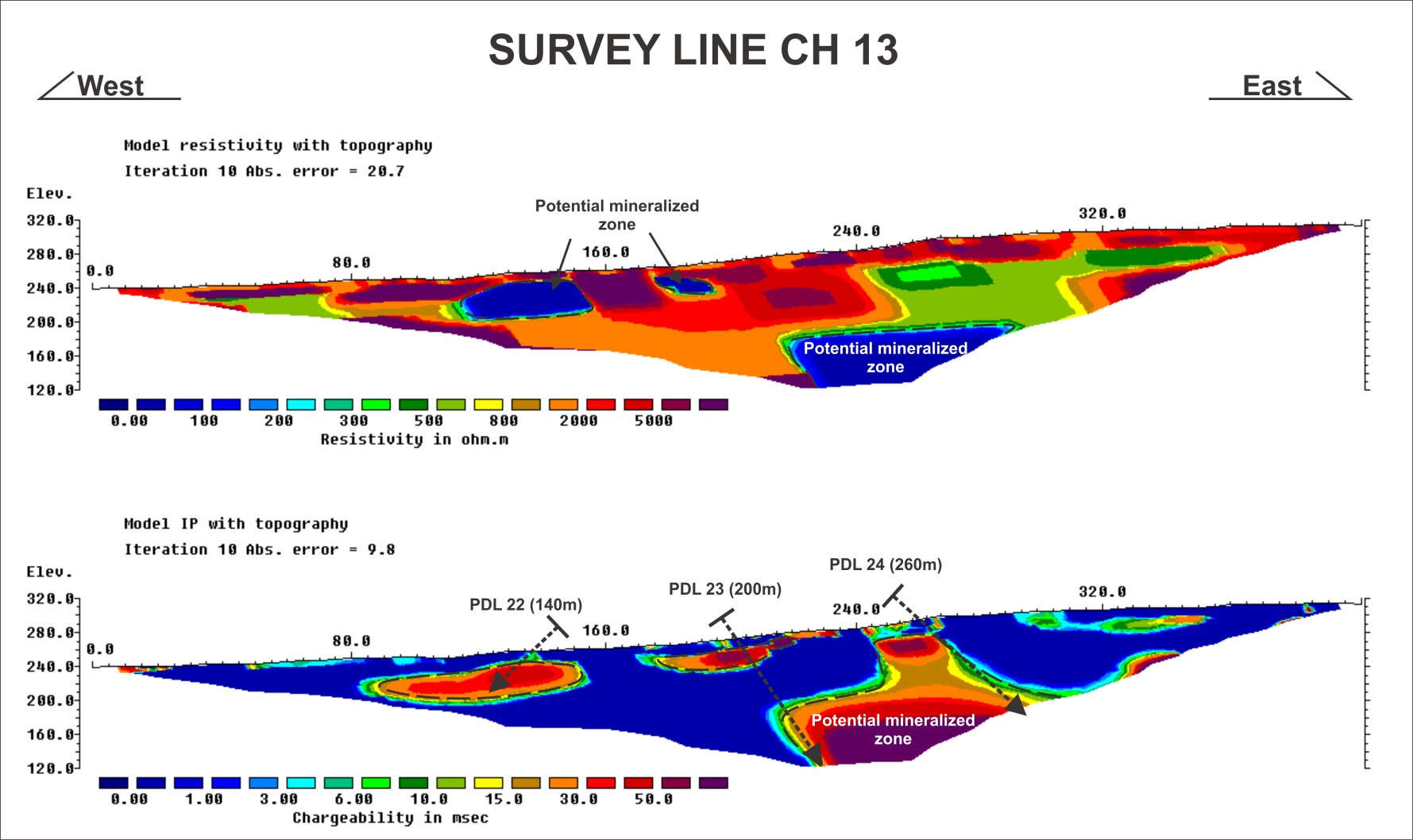

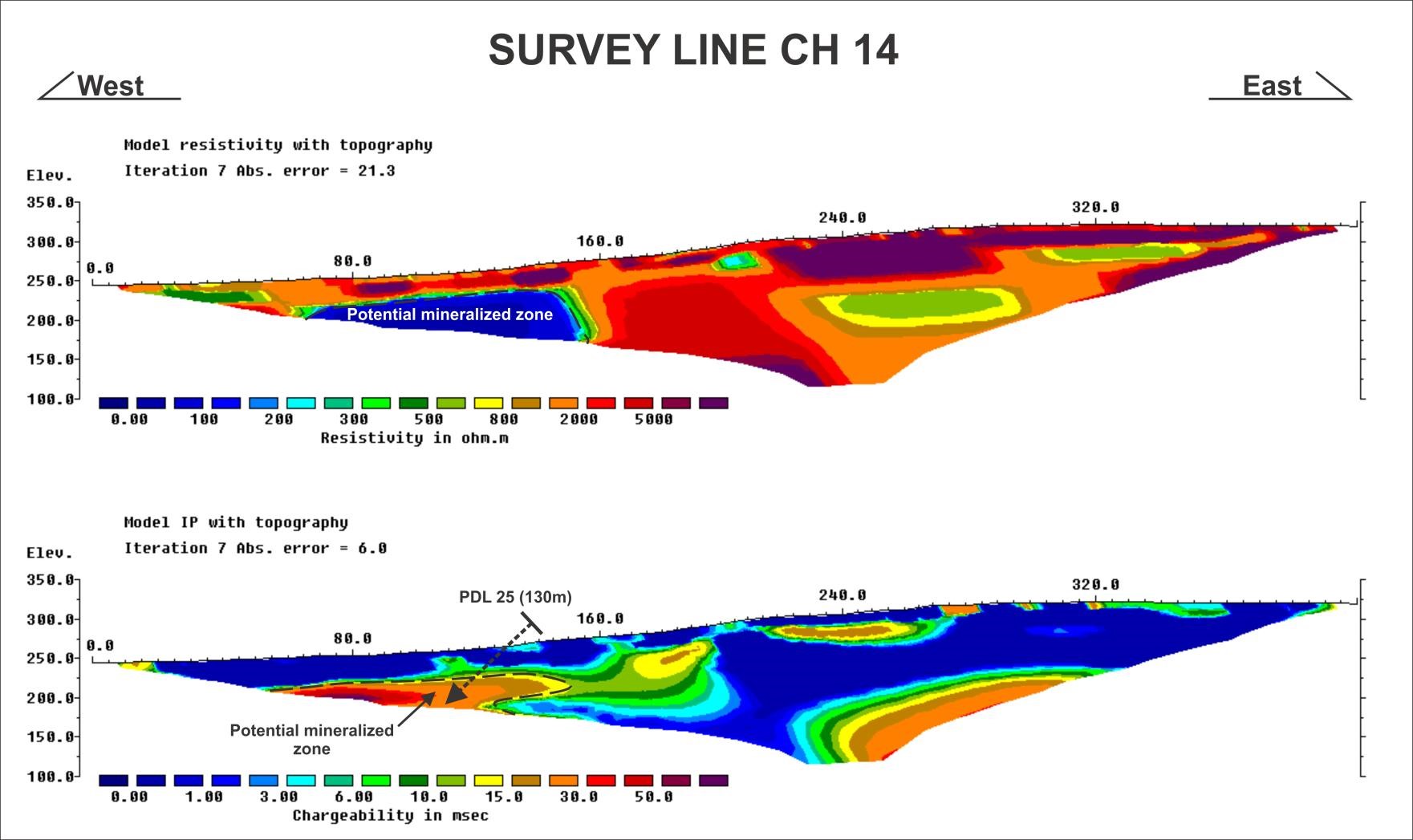

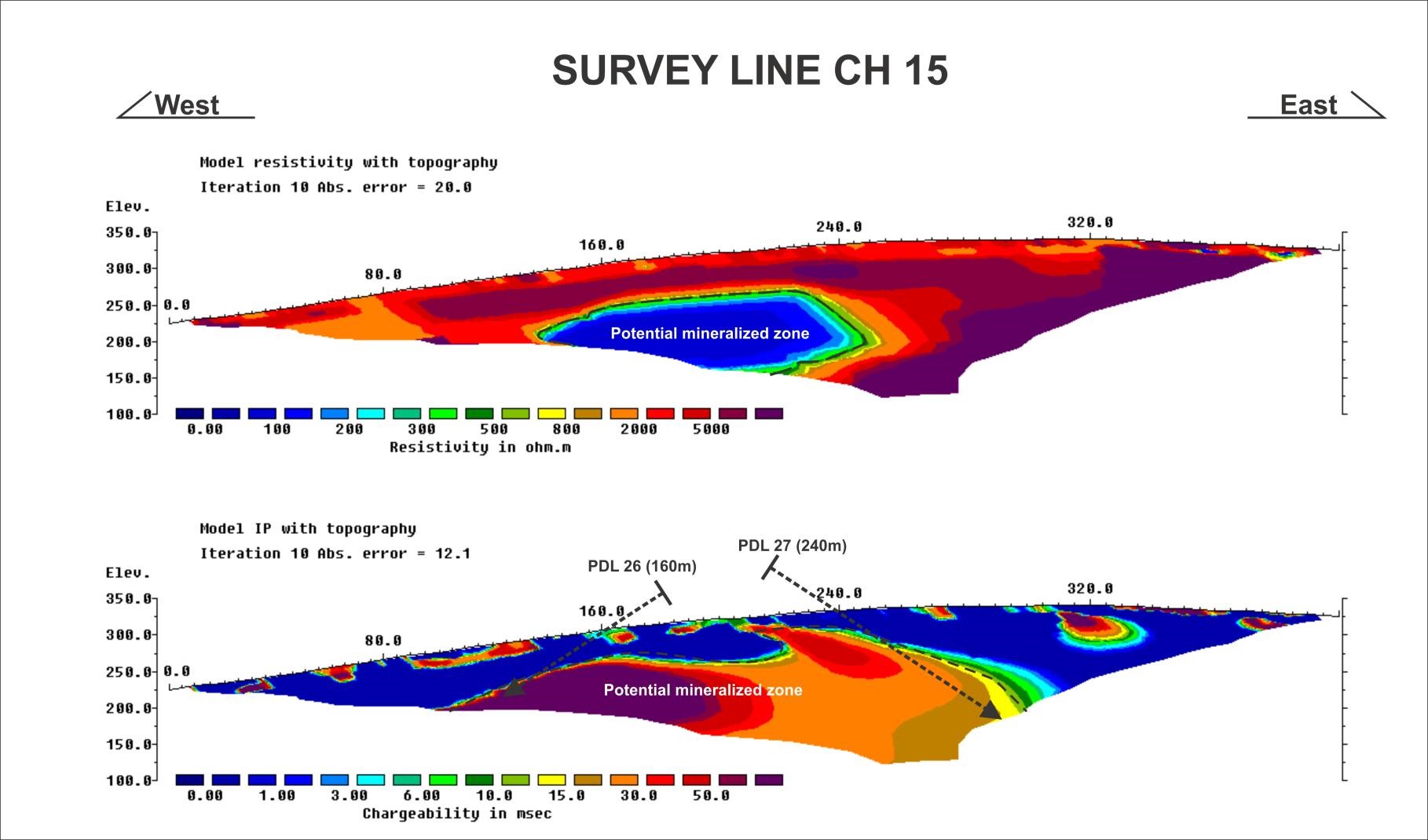

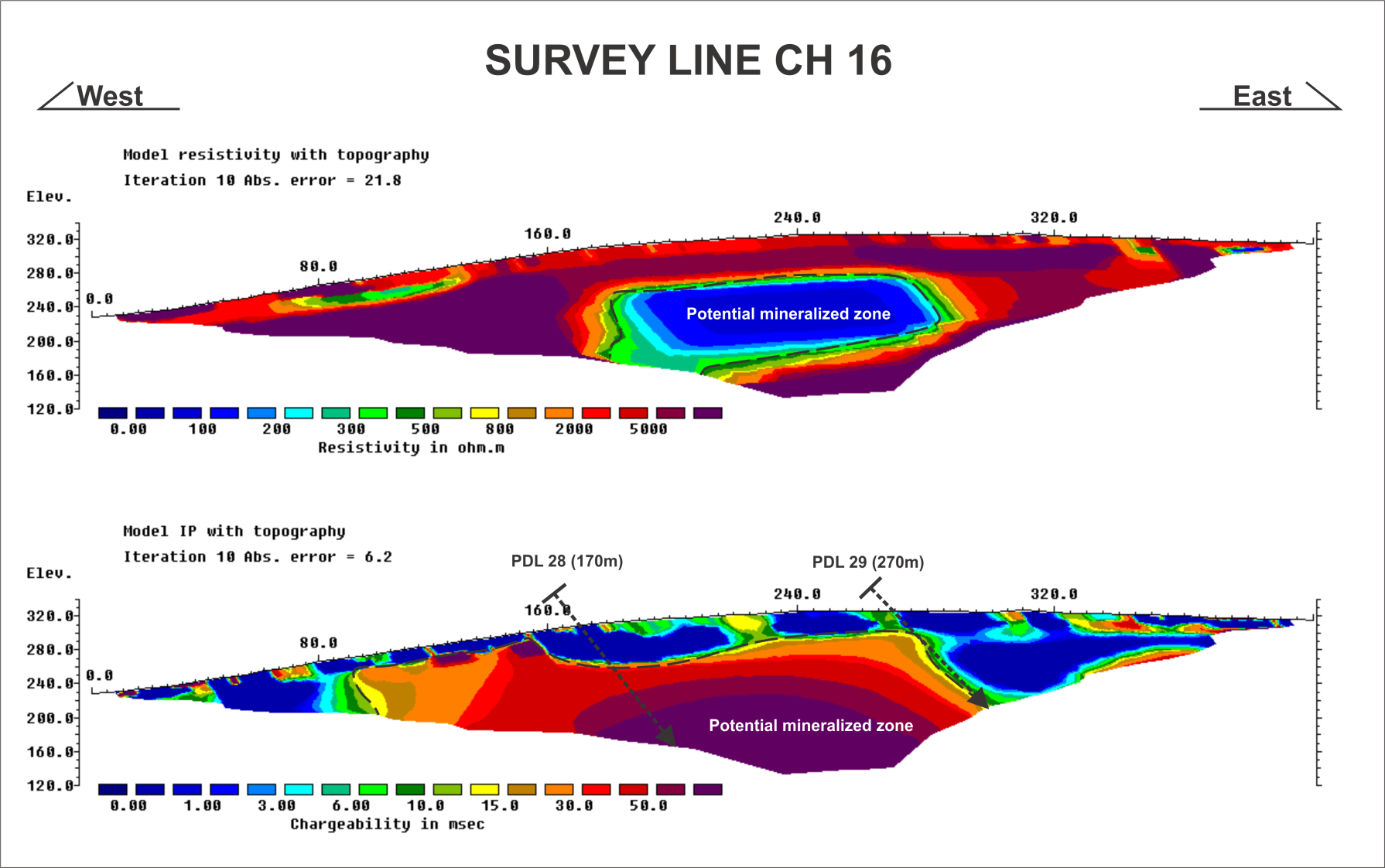

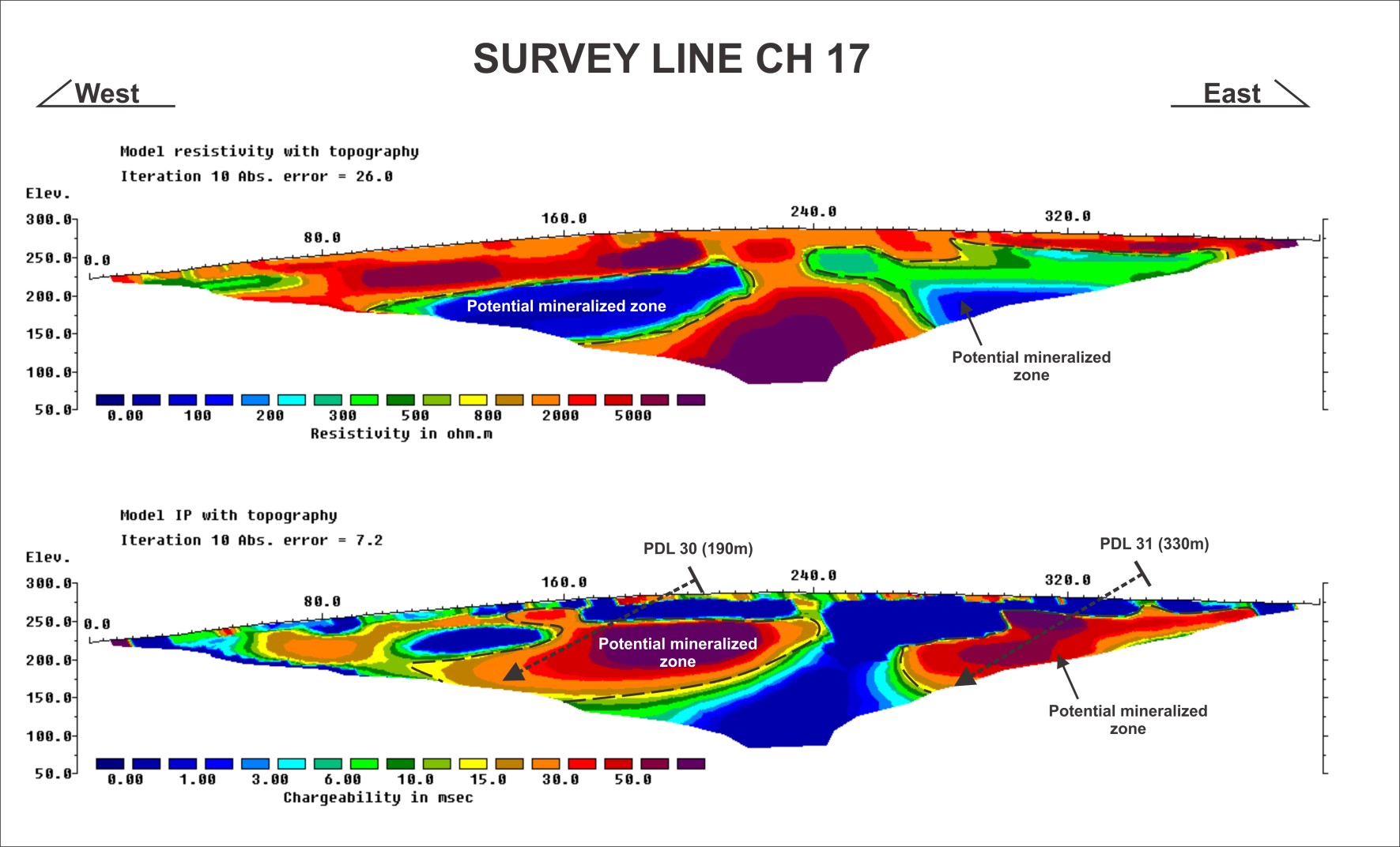

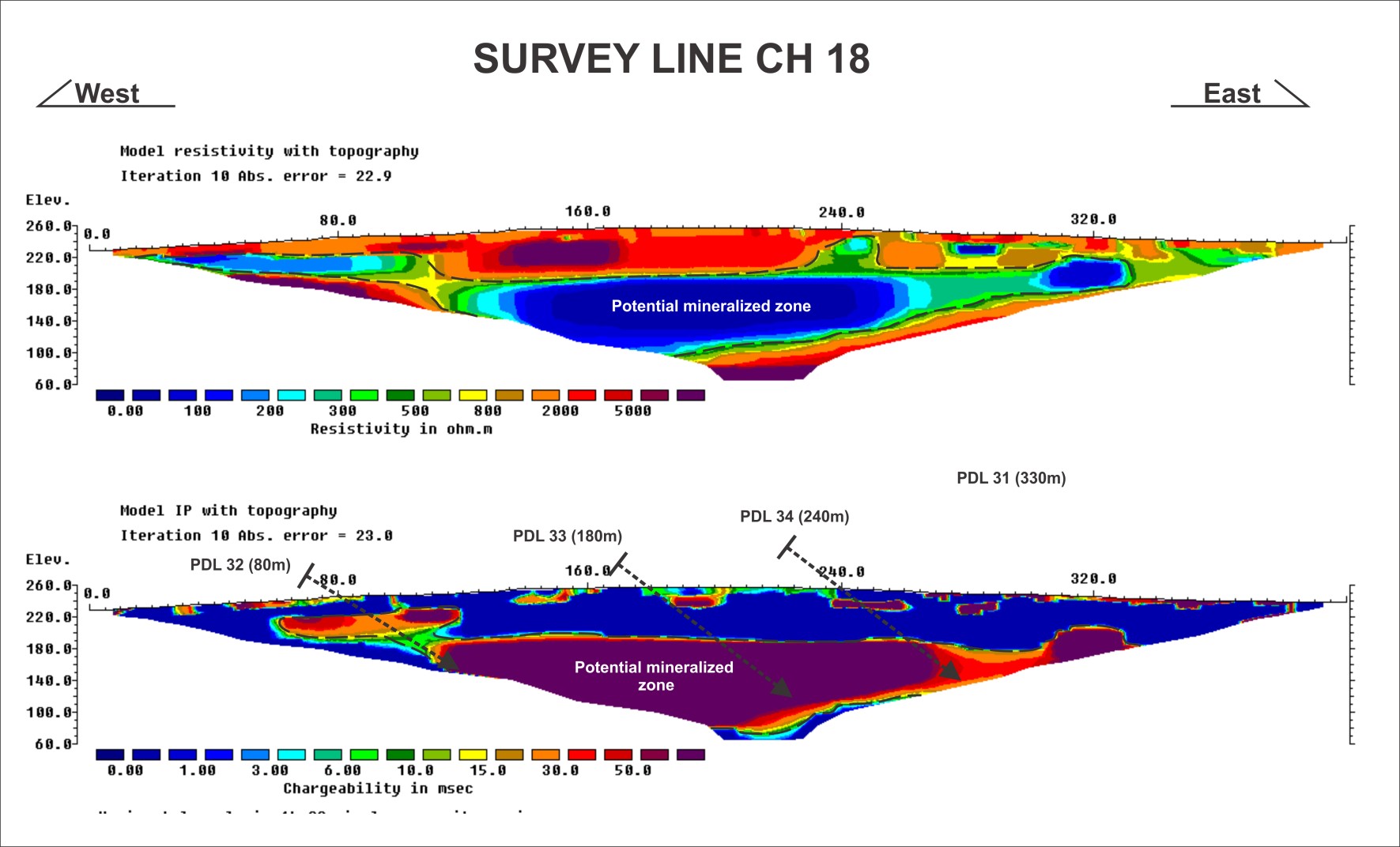

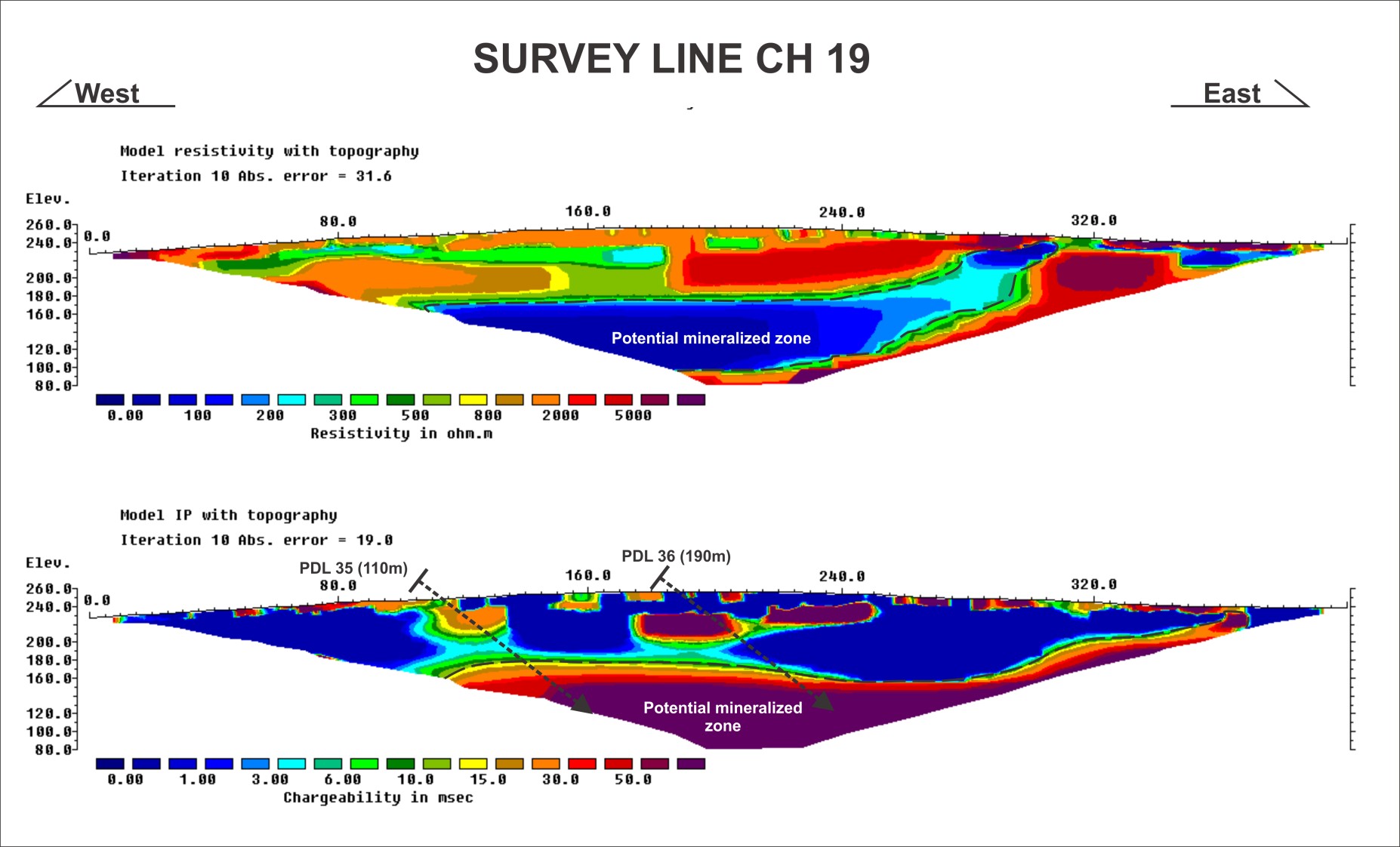

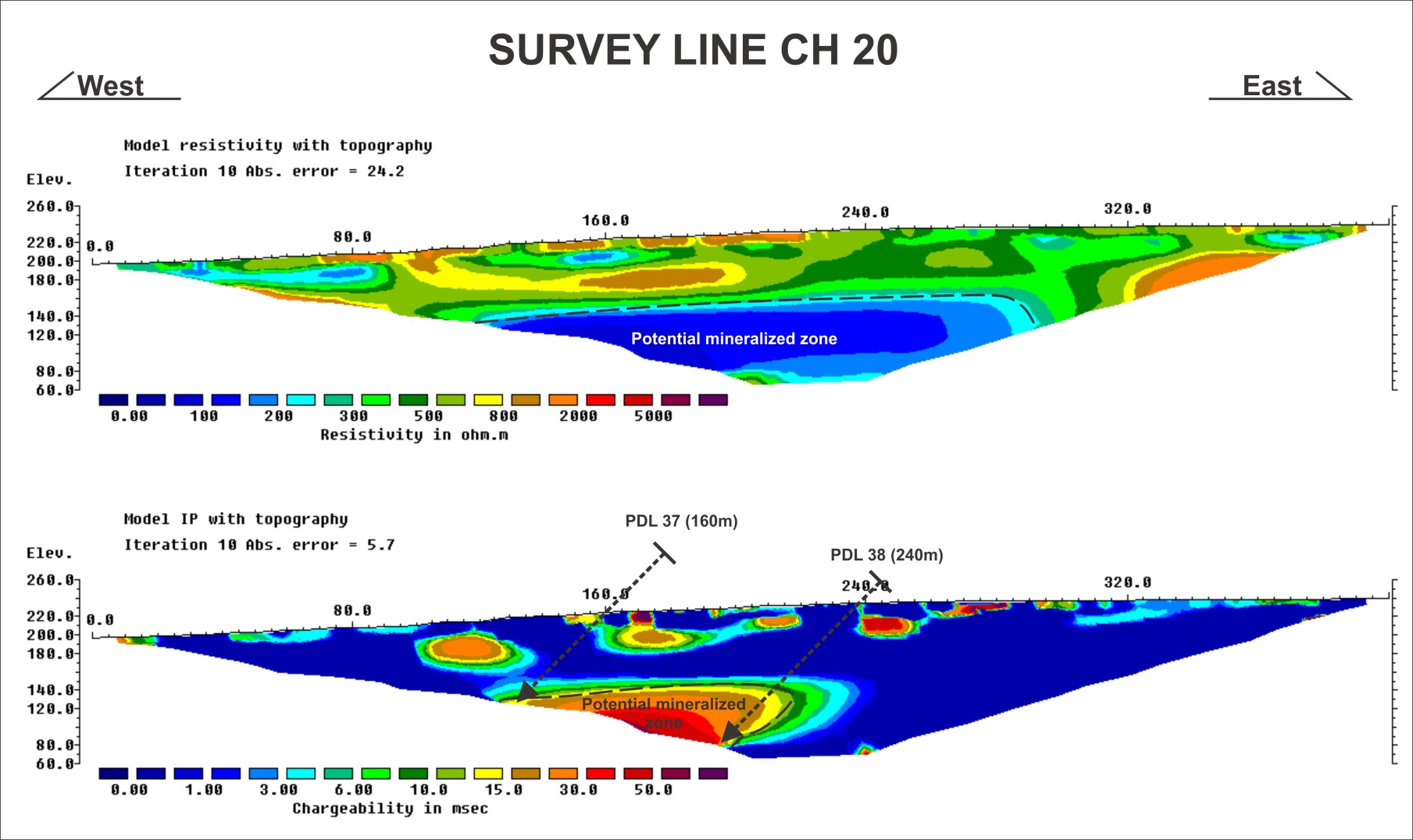

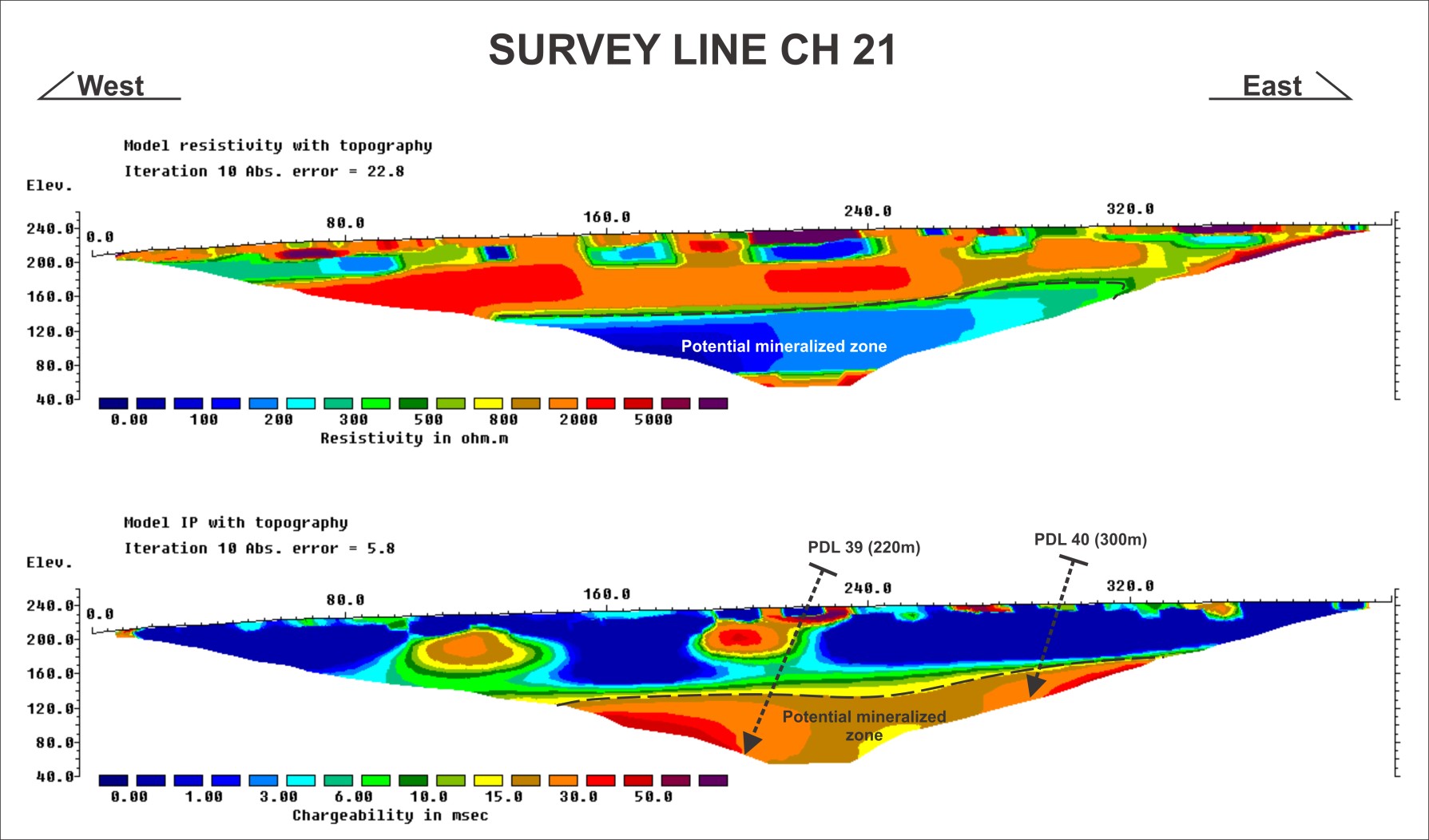

Supplement: Supplementary file 2 — Supplementary material [file mmc2.docx]
