# Supplementary material for: Data for the potential gold mineralization mapping with the applications of Electrical Resistivity Imaging and Induced Polarization geophysical surveys
Source: Data Brief. 2018 Dec 31;22:830–5. doi: 10.1016/j.dib.2018.12.086 (PMC6362863; doi:10.1016/j.dib.2018.12.086)
Supplement: Supplementary file 3 — Supplementary material [file mmc3.docx]

Survey Lines data

| **Survey Line** | **Distance (m)** | **Easting (m)** | **Northing (m)** | **Elevation (m)** |
| --- | --- | --- | --- | --- |
| CH 1 | 0 | 460484 | 553243 | 245 |
|  | 10 | 460497 | 553246 | 245 |
|  | 20 | 460505 | 553243 | 246 |
|  | 30 | 460516 | 553243 | 246 |
|  | 40 | 460526 | 553244 | 248 |
|  | 50 | 460536 | 553247 | 249 |
|  | 60 | 460544 | 553245 | 251 |
|  | 70 | 460553 | 553250 | 253 |
|  | 80 | 460562 | 553244 | 253 |
|  | 90 | 460572 | 553245 | 255 |
|  | 100 | 460581 | 553251 | 258 |
|  | 110 | 460589 | 553253 | 259 |
|  | 120 | 460597 | 553247 | 263 |
|  | 130 | 460606 | 553250 | 266 |
|  | 140 | 460617 | 553253 | 271 |
|  | 150 | 460625 | 553251 | 275 |
|  | 160 | 460635 | 553253 | 278 |
|  | 170 | 460646 | 553253 | 281 |
|  | 180 | 460655 | 553248 | 284 |
|  | 190 | 460664 | 553254 | 289 |
|  | 200 | 460683 | 553252 | 293 |
|  | 210 | 460693 | 553256 | 299 |
|  | 220 | 460703 | 553257 | 303 |
|  | 230 | 460715 | 553263 | 304 |
|  | 240 | 460724 | 553256 | 306 |
|  | 250 | 460736 | 553256 | 310 |
|  | 260 | 460749 | 553259 | 312 |
|  | 270 | 460764 | 553255 | 317 |
|  | 280 | 460774 | 553256 | 318 |
|  | 290 | 460787 | 553261 | 320 |
|  | 300 | 460798 | 553261 | 321 |
|  | 310 | 460807 | 553262 | 321 |
|  | 320 | 460817 | 553264 | 322 |
|  | 330 | 460828 | 553262 | 322 |
|  | 340 | 460837 | 553263 | 321 |
|  | 350 | 460848 | 553269 | 321 |
|  | 360 | 460859 | 553267 | 321 |
|  | 370 | 460865 | 553272 | 321 |
|  | 380 | 460872 | 553270 | 320 |
|  | 390 | 460877 | 553267 | 321 |
|  | 400 | 460884 | 553268 | 319 |

| **Survey Line** | **Distance (m)** | **Easting (m)** | **Northing (m)** | **Elevation (m)** |
| --- | --- | --- | --- | --- |
| CH 2 | 0 | 460488 | 553142 | 224 |
|  | 10 | 460494 | 553148 | 222 |
|  | 20 | 460501 | 553148 | 219 |
|  | 30 | 460510 | 553143 | 216 |
|  | 40 | 460520 | 553147 | 212 |
|  | 50 | 460530 | 553149 | 208 |
|  | 60 | 460540 | 553145 | 204 |
|  | 70 | 460550 | 553146 | 200 |
|  | 80 | 460561 | 553152 | 197 |
|  | 90 | 460570 | 553156 | 192 |
|  | 100 | 460581 | 553155 | 187 |
|  | 110 | 460591 | 553154 | 182 |
|  | 120 | 460600 | 553156 | 178 |
|  | 130 | 460608 | 553151 | 174 |
|  | 140 | 460618 | 553155 | 170 |
|  | 150 | 460627 | 553155 | 165 |
|  | 160 | 460637 | 553155 | 161 |
|  | 170 | 460647 | 553152 | 157 |
|  | 180 | 460659 | 553156 | 154 |
|  | 190 | 460667 | 553161 | 151 |
|  | 200 | 460685 | 553157 | 145 |
|  | 210 | 460695 | 553162 | 142 |
|  | 220 | 460705 | 553163 | 138 |
|  | 230 | 460716 | 553162 | 135 |
|  | 240 | 460728 | 553160 | 135 |
|  | 250 | 460739 | 553164 | 133 |
|  | 260 | 460747 | 553166 | 131 |
|  | 270 | 460758 | 553163 | 130 |
|  | 280 | 460767 | 553162 | 128 |
|  | 290 | 460776 | 553165 | 126 |
|  | 300 | 460786 | 553167 | 124 |
|  | 310 | 460798 | 553164 | 123 |
|  | 320 | 460810 | 553165 | 122 |
|  | 330 | 460822 | 553165 | 122 |
|  | 340 | 460833 | 553164 | 122 |
|  | 350 | 460843 | 553163 | 122 |
|  | 360 | 460853 | 553167 | 121 |
|  | 370 | 460865 | 553171 | 122 |
|  | 380 | 460874 | 553166 | 121 |
|  | 390 | 460883 | 553170 | 122 |
|  | 400 | 460887 | 553172 | 123 |

| **Survey Line** | **Distance (m)** | **Easting (m)** | **Northing (m)** | **Elevation (m)** |
| --- | --- | --- | --- | --- |
| CH 3 | 0 | 460482 | 553047 | 242 |
|  | 10 | 460489 | 553044 | 235 |
|  | 20 | 460497 | 553046 | 233 |
|  | 30 | 460506 | 553047 | 229 |
|  | 40 | 460514 | 553049 | 224 |
|  | 50 | 460524 | 553052 | 218 |
|  | 60 | 460534 | 553053 | 213 |
|  | 70 | 460545 | 553051 | 208 |
|  | 80 | 460558 | 553052 | 201 |
|  | 90 | 460567 | 553051 | 197 |
|  | 100 | 460576 | 553054 | 194 |
|  | 110 | 460586 | 553053 | 189 |
|  | 120 | 460595 | 553053 | 184 |
|  | 130 | 460605 | 553056 | 181 |
|  | 140 | 460616 | 553055 | 176 |
|  | 150 | 460625 | 553055 | 173 |
|  | 160 | 460634 | 553060 | 169 |
|  | 170 | 460642 | 553061 | 166 |
|  | 180 | 460652 | 553062 | 163 |
|  | 190 | 460663 | 553062 | 159 |
|  | 200 | 460674 | 553063 | 156 |
|  | 210 | 460686 | 553061 | 153 |
|  | 220 | 460696 | 553060 | 150 |
|  | 230 | 460707 | 553057 | 147 |
|  | 240 | 460718 | 553060 | 144 |
|  | 250 | 460728 | 553061 | 142 |
|  | 260 | 460739 | 553062 | 141 |
|  | 270 | 460748 | 553061 | 139 |
|  | 280 | 460759 | 553065 | 137 |
|  | 290 | 460770 | 553065 | 135 |
|  | 300 | 460781 | 553065 | 133 |
|  | 310 | 460791 | 553065 | 132 |
|  | 320 | 460805 | 553067 | 131 |
|  | 330 | 460814 | 553062 | 130 |
|  | 340 | 460824 | 553063 | 129 |
|  | 350 | 460835 | 553062 | 128 |
|  | 360 | 460845 | 553062 | 127 |
|  | 370 | 460855 | 553060 | 126 |
|  | 380 | 460866 | 553063 | 125 |
|  | 390 | 460878 | 553067 | 126 |
|  | 400 | 460884 | 553066 | 125 |

| **Survey Line** | **Distance (m)** | **Easting (m)** | **Northing (m)** | **Elevation (m)** |
| --- | --- | --- | --- | --- |
| CH 4 | 0 | 460472 | 552945 | 251 |
|  | 10 | 460483 | 552947 | 247 |
|  | 20 | 460491 | 552946 | 244 |
|  | 30 | 460502 | 552947 | 240 |
|  | 40 | 460510 | 552946 | 236 |
|  | 50 | 460520 | 552949 | 232 |
|  | 60 | 460530 | 552948 | 228 |
|  | 70 | 460540 | 552946 | 225 |
|  | 80 | 460552 | 552947 | 221 |
|  | 90 | 460561 | 552951 | 217 |
|  | 100 | 460571 | 552947 | 215 |
|  | 110 | 460587 | 552946 | 209 |
|  | 120 | 460594 | 552943 | 207 |
|  | 130 | 460604 | 552948 | 203 |
|  | 140 | 460614 | 552950 | 197 |
|  | 150 | 460624 | 552949 | 194 |
|  | 160 | 460636 | 552954 | 189 |
|  | 170 | 460650 | 552950 | 185 |
|  | 180 | 460657 | 552955 | 183 |
|  | 190 | 460667 | 552960 | 179 |
|  | 200 | 460693 | 552957 | 175 |
|  | 210 | 460674 | 552953 | 172 |
|  | 220 | 460704 | 552957 | 168 |
|  | 230 | 460712 | 552954 | 166 |
|  | 240 | 460725 | 552957 | 163 |
|  | 250 | 460735 | 552957 | 161 |
|  | 260 | 460747 | 552961 | 158 |
|  | 270 | 460757 | 552957 | 155 |
|  | 280 | 460769 | 552960 | 153 |
|  | 290 | 460778 | 552958 | 150 |
|  | 300 | 460787 | 552958 | 147 |
|  | 310 | 460797 | 552958 | 145 |
|  | 320 | 460806 | 552954 | 144 |
|  | 330 | 460818 | 552954 | 142 |
|  | 340 | 460828 | 552963 | 139 |
|  | 350 | 460838 | 552964 | 137 |
|  | 360 | 460856 | 552965 | 134 |
|  | 370 | 460847 | 552966 | 133 |
|  | 380 | 460864 | 552957 | 131 |
|  | 390 | 460876 | 552954 | 130 |
|  | 400 | 460883 | 552951 | 129 |

| **Survey Line** | **Distance (m)** | **Easting (m)** | **Northing (m)** | **Elevation (m)** |
| --- | --- | --- | --- | --- |
| CH 5 | 0 | 460487 | 552858 | 266 |
|  | 10 | 460494 | 552859 | 264 |
|  | 20 | 460501 | 552859 | 262 |
|  | 30 | 460507 | 552859 | 260 |
|  | 40 | 460518 | 552857 | 256 |
|  | 50 | 460528 | 552855 | 253 |
|  | 60 | 460539 | 552856 | 248 |
|  | 70 | 460549 | 552856 | 244 |
|  | 80 | 460557 | 552856 | 241 |
|  | 90 | 460566 | 552859 | 237 |
|  | 100 | 460575 | 552863 | 233 |
|  | 110 | 460588 | 552862 | 229 |
|  | 120 | 460599 | 552861 | 227 |
|  | 130 | 460610 | 552861 | 222 |
|  | 140 | 460620 | 552861 | 217 |
|  | 150 | 460630 | 552857 | 215 |
|  | 160 | 460641 | 552860 | 210 |
|  | 170 | 460651 | 552861 | 207 |
|  | 180 | 460661 | 552861 | 203 |
|  | 190 | 460671 | 552863 | 199 |
|  | 200 | 460681 | 552857 | 196 |
|  | 210 | 460690 | 552860 | 193 |
|  | 220 | 460701 | 552855 | 188 |
|  | 230 | 460713 | 552856 | 183 |
|  | 240 | 460724 | 552856 | 180 |
|  | 250 | 460735 | 552858 | 177 |
|  | 260 | 460746 | 552859 | 173 |
|  | 270 | 460757 | 552857 | 169 |
|  | 280 | 460767 | 552858 | 165 |
|  | 290 | 460775 | 552855 | 163 |
|  | 300 | 460787 | 552858 | 159 |
|  | 310 | 460798 | 552857 | 154 |
|  | 320 | 460811 | 552860 | 151 |
|  | 330 | 460822 | 552858 | 147 |
|  | 340 | 460833 | 552859 | 145 |
|  | 350 | 460843 | 552857 | 142 |
|  | 360 | 460854 | 552858 | 139 |
|  | 370 | 460865 | 552858 | 136 |
|  | 380 | 460874 | 552857 | 135 |
|  | 390 | 460884 | 552858 | 134 |
|  | 400 | 460894 | 552861 | 132 |

| **Survey Line** | **Distance (m)** | **Easting (m)** | **Northing (m)** | **Elevation (m)** |
| --- | --- | --- | --- | --- |
| CH 6 | 0 | 460499 | 552760 | 293 |
|  | 10 | 460505 | 552762 | 290 |
|  | 20 | 460516 | 552761 | 286 |
|  | 30 | 460527 | 552762 | 283 |
|  | 40 | 460536 | 552761 | 280 |
|  | 50 | 460546 | 552763 | 276 |
|  | 60 | 460556 | 552760 | 274 |
|  | 70 | 460567 | 552756 | 270 |
|  | 80 | 460574 | 552755 | 266 |
|  | 90 | 460585 | 552755 | 261 |
|  | 100 | 460594 | 552759 | 255 |
|  | 110 | 460605 | 552759 | 249 |
|  | 120 | 460614 | 552760 | 244 |
|  | 130 | 460623 | 552759 | 239 |
|  | 140 | 460636 | 552765 | 230 |
|  | 150 | 460645 | 552761 | 228 |
|  | 160 | 460654 | 552764 | 222 |
|  | 170 | 460666 | 552754 | 219 |
|  | 180 | 460676 | 552754 | 214 |
|  | 190 | 460686 | 552754 | 209 |
|  | 200 | 460696 | 552752 | 204 |
|  | 210 | 460708 | 552749 | 199 |
|  | 220 | 460717 | 552751 | 194 |
|  | 230 | 460727 | 552752 | 190 |
|  | 240 | 460737 | 552752 | 186 |
|  | 250 | 460748 | 552754 | 183 |
|  | 260 | 460757 | 552754 | 179 |
|  | 270 | 460768 | 552754 | 175 |
|  | 280 | 460778 | 552755 | 172 |
|  | 290 | 460786 | 552754 | 169 |
|  | 300 | 460796 | 552750 | 166 |
|  | 310 | 460806 | 552750 | 163 |
|  | 320 | 460816 | 552749 | 161 |
|  | 330 | 460826 | 552752 | 158 |
|  | 340 | 460836 | 552750 | 154 |
|  | 350 | 460844 | 552750 | 153 |
|  | 360 | 460855 | 552746 | 150 |
|  | 370 | 460866 | 552746 | 148 |
|  | 380 | 460877 | 552745 | 147 |
|  | 390 | 460889 | 552745 | 146 |
|  | 400 | 460898 | 552748 | 145 |

| **Survey Line** | **Distance (m)** | **Easting (m)** | **Northing (m)** | **Elevation (m)** |
| --- | --- | --- | --- | --- |
| CH 7 | 0 | 460495 | 552666 | 309 |
|  | 10 | 460505 | 552663 | 307 |
|  | 20 | 460514 | 552660 | 305 |
|  | 30 | 460526 | 552660 | 301 |
|  | 40 | 460535 | 552665 | 298 |
|  | 50 | 460543 | 552659 | 296 |
|  | 60 | 460553 | 552651 | 293 |
|  | 70 | 460563 | 552650 | 290 |
|  | 80 | 460574 | 552650 | 284 |
|  | 90 | 460585 | 552651 | 277 |
|  | 100 | 460594 | 552651 | 272 |
|  | 110 | 460609 | 552652 | 264 |
|  | 120 | 460618 | 552655 | 260 |
|  | 130 | 460626 | 552654 | 256 |
|  | 140 | 460635 | 552656 | 252 |
|  | 150 | 460645 | 552656 | 245 |
|  | 160 | 460658 | 552654 | 241 |
|  | 170 | 460666 | 552654 | 235 |
|  | 180 | 460679 | 552652 | 229 |
|  | 190 | 460690 | 552655 | 222 |
|  | 200 | 460698 | 552648 | 219 |
|  | 210 | 460709 | 552650 | 213 |
|  | 220 | 460720 | 552655 | 209 |
|  | 230 | 460731 | 552652 | 206 |
|  | 240 | 460740 | 552648 | 203 |
|  | 250 | 460748 | 552651 | 199 |
|  | 260 | 460759 | 552648 | 197 |
|  | 270 | 460768 | 552651 | 195 |
|  | 280 | 460778 | 552650 | 190 |
|  | 290 | 460789 | 552651 | 187 |
|  | 300 | 460799 | 552650 | 186 |
|  | 310 | 460807 | 552650 | 186 |
|  | 320 | 460817 | 552651 | 184 |
|  | 330 | 460826 | 552650 | 183 |
|  | 340 | 460836 | 552650 | 182 |
|  | 350 | 460845 | 552648 | 182 |
|  | 360 | 460855 | 552646 | 182 |
|  | 370 | 460866 | 552643 | 182 |
|  | 380 | 460875 | 552643 | 180 |
|  | 390 | 460884 | 552643 | 180 |
|  | 400 | 460893 | 552642 | 180 |

| **Survey Line** | **Distance (m)** | **Easting (m)** | **Northing (m)** | **Elevation (m)** |
| --- | --- | --- | --- | --- |
| CH 8 | 0 | 460498 | 552552 | 320 |
|  | 10 | 460507 | 552553 | 317 |
|  | 20 | 460517 | 552551 | 313 |
|  | 30 | 460525 | 552553 | 310 |
|  | 40 | 460535 | 552556 | 306 |
|  | 50 | 460545 | 552558 | 302 |
|  | 60 | 460553 | 552558 | 229 |
|  | 70 | 460563 | 552561 | 295 |
|  | 80 | 460572 | 552561 | 290 |
|  | 90 | 460585 | 552560 | 285 |
|  | 100 | 460593 | 552557 | 281 |
|  | 110 | 460604 | 552559 | 275 |
|  | 120 | 460614 | 552559 | 273 |
|  | 130 | 460624 | 552561 | 269 |
|  | 140 | 460633 | 552547 | 267 |
|  | 150 | 460647 | 552550 | 261 |
|  | 160 | 460657 | 552550 | 255 |
|  | 170 | 460668 | 552552 | 255 |
|  | 180 | 460678 | 552553 | 244 |
|  | 190 | 460688 | 552556 | 240 |
|  | 200 | 460698 | 552556 | 235 |
|  | 210 | 460707 | 552555 | 231 |
|  | 220 | 460717 | 552554 | 227 |
|  | 230 | 460730 | 552562 | 223 |
|  | 240 | 460739 | 552557 | 221 |
|  | 250 | 460747 | 552559 | 218 |
|  | 260 | 460757 | 552560 | 216 |
|  | 270 | 460768 | 552559 | 214 |
|  | 280 | 460778 | 552561 | 210 |
|  | 290 | 460786 | 552560 | 208 |
|  | 300 | 460798 | 552558 | 208 |
|  | 310 | 460806 | 552552 | 208 |
|  | 320 | 460816 | 552555 | 208 |
|  | 330 | 460827 | 552554 | 208 |
|  | 340 | 460835 | 552554 | 208 |
|  | 350 | 460845 | 552552 | 210 |
|  | 360 | 460855 | 552552 | 209 |
|  | 370 | 460864 | 552554 | 209 |
|  | 380 | 460875 | 552555 | 209 |
|  | 390 | 460885 | 552556 | 209 |
|  | 400 | 460895 | 552557 | 207 |

| **Survey Line** | **Distance (m)** | **Easting (m)** | **Northing (m)** | **Elevation (m)** |
| --- | --- | --- | --- | --- |
| CH 9 | 0 | 460485 | 552458 | 314 |
|  | 10 | 460491 | 552458 | 310 |
|  | 20 | 460499 | 552458 | 308 |
|  | 30 | 460507 | 552458 | 306 |
|  | 40 | 460518 | 552458 | 303 |
|  | 50 | 460526 | 552457 | 299 |
|  | 60 | 460537 | 552456 | 297 |
|  | 70 | 460546 | 552456 | 294 |
|  | 80 | 460557 | 552455 | 290 |
|  | 90 | 460567 | 552455 | 288 |
|  | 100 | 460579 | 552453 | 287 |
|  | 110 | 460589 | 552455 | 284 |
|  | 120 | 460597 | 552456 | 282 |
|  | 130 | 460606 | 552455 | 279 |
|  | 140 | 460615 | 552456 | 276 |
|  | 150 | 460624 | 552455 | 273 |
|  | 160 | 460634 | 552455 | 271 |
|  | 170 | 460643 | 552455 | 268 |
|  | 180 | 460653 | 552453 | 263 |
|  | 190 | 460661 | 552452 | 253 |
|  | 200 | 460673 | 552454 | 257 |
|  | 210 | 460682 | 552453 | 253 |
|  | 220 | 460694 | 552452 | 247 |
|  | 230 | 460705 | 552452 | 243 |
|  | 240 | 460714 | 552452 | 240 |
|  | 250 | 460727 | 552452 | 235 |
|  | 260 | 460735 | 552452 | 233 |
|  | 270 | 460748 | 552451 | 231 |
|  | 280 | 460761 | 552451 | 229 |
|  | 290 | 460774 | 552451 | 228 |
|  | 300 | 460787 | 552451 | 227 |
|  | 310 | 460801 | 552451 | 224 |
|  | 320 | 460810 | 552452 | 225 |
|  | 330 | 460820 | 552451 | 226 |
|  | 340 | 460827 | 552452 | 226 |
|  | 350 | 460837 | 552452 | 226 |
|  | 360 | 460847 | 552451 | 227 |
|  | 370 | 460858 | 552450 | 227 |
|  | 380 | 460867 | 552450 | 229 |
|  | 390 | 460876 | 552449 | 229 |
|  | 400 | 460886 | 552450 | 229 |

| **Survey Line** | **Distance (m)** | **Easting (m)** | **Northing (m)** | **Elevation (m)** |
| --- | --- | --- | --- | --- |
| CH 10 | 0 | 460503 | 552354 | 272 |
|  | 10 | 460514 | 552351 | 272 |
|  | 20 | 460523 | 552355 | 273 |
|  | 30 | 460533 | 552353 | 268 |
|  | 40 | 460541 | 552351 | 266 |
|  | 50 | 460551 | 552355 | 266 |
|  | 60 | 460560 | 552354 | 264 |
|  | 70 | 460570 | 552354 | 264 |
|  | 80 | 460577 | 552355 | 266 |
|  | 90 | 460586 | 552354 | 257 |
|  | 100 | 460594 | 552355 | 252 |
|  | 110 | 460604 | 552357 | 255 |
|  | 120 | 460615 | 552352 | 253 |
|  | 130 | 460620 | 552357 | 253 |
|  | 140 | 460626 | 552355 | 252 |
|  | 150 | 460632 | 552354 | 249 |
|  | 160 | 460637 | 552356 | 249 |
|  | 170 | 460646 | 552353 | 246 |
|  | 180 | 460658 | 552358 | 245 |
|  | 190 | 460667 | 552356 | 243 |
|  | 200 | 460676 | 552350 | 241 |
|  | 210 | 460688 | 552354 | 241 |
|  | 220 | 460699 | 552355 | 241 |
|  | 230 | 460712 | 552356 | 241 |
|  | 240 | 460721 | 552353 | 241 |
|  | 250 | 460730 | 552352 | 240 |
|  | 260 | 460738 | 552355 | 239 |
|  | 270 | 460748 | 552352 | 238 |
|  | 280 | 460755 | 552359 | 237 |
|  | 290 | 460762 | 552356 | 238 |
|  | 300 | 460770 | 552354 | 237 |
|  | 310 | 460775 | 552354 | 235 |
|  | 320 | 460782 | 552355 | 237 |
|  | 330 | 460791 | 552355 | 238 |
|  | 340 | 460802 | 552355 | 239 |
|  | 350 | 460814 | 552351 | 241 |
|  | 360 | 460828 | 552355 | 239 |
|  | 370 | 460844 | 552355 | 242 |
|  | 380 | 460859 | 552349 | 243 |
|  | 390 | 460876 | 552355 | 244 |
|  | 400 | 460889 | 552356 | 246 |

| **Survey Line** | **Distance (m)** | **Easting (m)** | **Northing (m)** | **Elevation (m)** |
| --- | --- | --- | --- | --- |
| CH 11 | 0 | 460488 | 552255 | 240 |
|  | 10 | 460499 | 552257 | 240 |
|  | 20 | 460511 | 552256 | 239 |
|  | 30 | 460520 | 552258 | 240 |
|  | 40 | 460531 | 552258 | 241 |
|  | 50 | 460539 | 552262 | 241 |
|  | 60 | 460552 | 552261 | 242 |
|  | 70 | 460562 | 552258 | 242 |
|  | 80 | 460569 | 552257 | 242 |
|  | 90 | 460575 | 552255 | 242 |
|  | 100 | 460582 | 552246 | 242 |
|  | 110 | 460590 | 552254 | 242 |
|  | 120 | 460603 | 552250 | 243 |
|  | 130 | 460608 | 552253 | 243 |
|  | 140 | 460619 | 552249 | 243 |
|  | 150 | 460629 | 552251 | 244 |
|  | 160 | 460639 | 552253 | 245 |
|  | 170 | 460651 | 552252 | 245 |
|  | 180 | 460655 | 552251 | 246 |
|  | 190 | 460665 | 552251 | 247 |
|  | 200 | 460676 | 552250 | 248 |
|  | 210 | 460687 | 552250 | 251 |
|  | 220 | 460694 | 552247 | 251 |
|  | 230 | 460705 | 552251 | 252 |
|  | 240 | 460717 | 552253 | 254 |
|  | 250 | 460726 | 552251 | 254 |
|  | 260 | 460732 | 552254 | 254 |
|  | 270 | 460740 | 552244 | 256 |
|  | 280 | 460748 | 552250 | 255 |
|  | 290 | 460758 | 552249 | 257 |
|  | 300 | 460768 | 552248 | 261 |
|  | 310 | 460774 | 552248 | 260 |
|  | 320 | 460783 | 552249 | 261 |
|  | 330 | 460792 | 552244 | 266 |
|  | 340 | 460798 | 552252 | 263 |
|  | 350 | 460810 | 552252 | 264 |
|  | 360 | 460822 | 552247 | 266 |
|  | 370 | 460840 | 552244 | 270 |
|  | 380 | 460852 | 552253 | 267 |
|  | 390 | 460867 | 552245 | 272 |
|  | 400 | 460874 | 552246 | 273 |

| **Survey Line** | **Distance (m)** | **Easting (m)** | **Northing (m)** | **Elevation (m)** |
| --- | --- | --- | --- | --- |
| CH 12 | 0 | 460484 | 552150 | 237 |
|  | 10 | 460492 | 552158 | 238 |
|  | 20 | 460499 | 552154 | 238 |
|  | 30 | 460506 | 552158 | 240 |
|  | 40 | 460519 | 552156 | 242 |
|  | 50 | 460523 | 552150 | 243 |
|  | 60 | 460532 | 552156 | 245 |
|  | 70 | 460543 | 552151 | 247 |
|  | 80 | 460554 | 552152 | 249 |
|  | 90 | 460562 | 552150 | 250 |
|  | 100 | 460577 | 552151 | 250 |
|  | 110 | 460584 | 552156 | 250 |
|  | 120 | 460594 | 552157 | 250 |
|  | 130 | 460606 | 552155 | 252 |
|  | 140 | 460618 | 552155 | 252 |
|  | 150 | 460631 | 552158 | 251 |
|  | 160 | 460640 | 552151 | 255 |
|  | 170 | 460650 | 552155 | 255 |
|  | 180 | 460659 | 552156 | 257 |
|  | 190 | 460672 | 552157 | 259 |
|  | 200 | 460682 | 552158 | 261 |
|  | 210 | 460691 | 552153 | 263 |
|  | 220 | 460701 | 552158 | 264 |
|  | 230 | 460712 | 552157 | 266 |
|  | 240 | 460725 | 552157 | 268 |
|  | 250 | 460738 | 552153 | 266 |
|  | 260 | 460747 | 552159 | 271 |
|  | 270 | 460751 | 552158 | 274 |
|  | 280 | 460763 | 552156 | 274 |
|  | 290 | 460773 | 552160 | 279 |
|  | 300 | 460783 | 552154 | 284 |
|  | 310 | 460796 | 552157 | 284 |
|  | 320 | 460809 | 552159 | 285 |
|  | 330 | 460820 | 552159 | 288 |
|  | 340 | 460827 | 552153 | 292 |
|  | 350 | 460832 | 552159 | 292 |
|  | 360 | 460841 | 552158 | 293 |
|  | 370 | 460849 | 552161 | 293 |
|  | 380 | 460853 | 552154 | 298 |
|  | 390 | 460865 | 552155 | 300 |
|  | 400 | 460876 | 552160 | 299 |

| **Survey Line** | **Distance (m)** | **Easting (m)** | **Northing (m)** | **Elevation (m)** |
| --- | --- | --- | --- | --- |
| CH 13 | 0 | 460481 | 552048 | 240 |
|  | 10 | 460492 | 552054 | 241 |
|  | 20 | 460500 | 552050 | 241 |
|  | 30 | 460512 | 552058 | 242 |
|  | 40 | 460520 | 552048 | 244 |
|  | 50 | 460529 | 552055 | 244 |
|  | 60 | 460545 | 552056 | 247 |
|  | 70 | 460553 | 552054 | 248 |
|  | 80 | 460564 | 552058 | 250 |
|  | 90 | 460573 | 552056 | 251 |
|  | 100 | 460581 | 552057 | 252 |
|  | 110 | 460589 | 552051 | 25 |
|  | 120 | 460597 | 552056 | 255 |
|  | 130 | 460604 | 552052 | 258 |
|  | 140 | 460613 | 552059 | 259 |
|  | 150 | 460620 | 552058 | 261 |
|  | 160 | 460628 | 552060 | 262 |
|  | 170 | 460643 | 552056 | 264 |
|  | 180 | 460655 | 552052 | 266 |
|  | 190 | 460665 | 552052 | 269 |
|  | 200 | 460685 | 552051 | 273 |
|  | 210 | 460698 | 552056 | 277 |
|  | 220 | 460708 | 552056 | 281 |
|  | 230 | 460721 | 552057 | 283 |
|  | 240 | 460731 | 552058 | 285 |
|  | 250 | 460747 | 552059 | 289 |
|  | 260 | 460754 | 552048 | 295 |
|  | 270 | 460765 | 552055 | 300 |
|  | 280 | 460772 | 552056 | 300 |
|  | 290 | 460782 | 552054 | 303 |
|  | 300 | 460789 | 552053 | 305 |
|  | 310 | 460799 | 552056 | 307 |
|  | 320 | 460806 | 552050 | 307 |
|  | 330 | 460814 | 552055 | 310 |
|  | 340 | 460823 | 552057 | 310 |
|  | 350 | 460829 | 552052 | 310 |
|  | 360 | 460837 | 552057 | 313 |
|  | 370 | 460843 | 552056 | 313 |
|  | 380 | 460850 | 552056 | 314 |
|  | 390 | 460861 | 552057 | 315 |
|  | 400 | 460870 | 552057 | 314 |

| **Survey Line** | **Distance (m)** | **Easting (m)** | **Northing (m)** | **Elevation (m)** |
| --- | --- | --- | --- | --- |
| CH 14 | 0 | 460476 | 551942 | 245 |
|  | 10 | 460485 | 551941 | 245 |
|  | 20 | 460492 | 551942 | 246 |
|  | 30 | 460499 | 551943 | 246 |
|  | 40 | 460510 | 551947 | 248 |
|  | 50 | 460521 | 551947 | 249 |
|  | 60 | 460531 | 551943 | 251 |
|  | 70 | 460545 | 551944 | 253 |
|  | 80 | 460555 | 551940 | 253 |
|  | 90 | 460570 | 551941 | 255 |
|  | 100 | 460579 | 551943 | 258 |
|  | 110 | 460585 | 551950 | 259 |
|  | 120 | 460595 | 551950 | 263 |
|  | 130 | 460607 | 551951 | 266 |
|  | 140 | 460621 | 551950 | 271 |
|  | 150 | 460636 | 551949 | 275 |
|  | 160 | 460643 | 551955 | 278 |
|  | 170 | 460656 | 551952 | 281 |
|  | 180 | 460667 | 551955 | 284 |
|  | 190 | 460680 | 551950 | 289 |
|  | 200 | 460691 | 551961 | 293 |
|  | 210 | 460707 | 551952 | 299 |
|  | 220 | 460717 | 551954 | 303 |
|  | 230 | 460724 | 551956 | 304 |
|  | 240 | 460733 | 551957 | 306 |
|  | 250 | 460742 | 551953 | 310 |
|  | 260 | 460752 | 551954 | 312 |
|  | 270 | 460760 | 551955 | 317 |
|  | 280 | 460770 | 551945 | 318 |
|  | 290 | 460776 | 551956 | 320 |
|  | 300 | 460784 | 551956 | 321 |
|  | 310 | 460790 | 551955 | 321 |
|  | 320 | 460797 | 551952 | 322 |
|  | 330 | 460807 | 551953 | 322 |
|  | 340 | 460818 | 551957 | 321 |
|  | 350 | 460825 | 551954 | 321 |
|  | 360 | 460837 | 551955 | 321 |
|  | 370 | 460847 | 551954 | 321 |
|  | 380 | 460854 | 551958 | 320 |
|  | 390 | 460862 | 551956 | 321 |
|  | 400 | 460872 | 551957 | 319 |

| **Survey Line** | **Distance (m)** | **Easting (m)** | **Northing (m)** | **Elevation (m)** |
| --- | --- | --- | --- | --- |
| CH 15 | 0 | 460079 | 552541 | 225 |
|  | 10 | 460089 | 552542 | 231 |
|  | 20 | 460099 | 552542 | 234 |
|  | 30 | 460109 | 552542 | 239 |
|  | 40 | 460119 | 552543 | 244 |
|  | 50 | 460129 | 552543 | 250 |
|  | 60 | 460139 | 552543 | 255 |
|  | 70 | 460149 | 552544 | 261 |
|  | 80 | 460159 | 552544 | 267 |
|  | 90 | 460169 | 552545 | 272 |
|  | 100 | 460179 | 552545 | 276 |
|  | 110 | 460189 | 552545 | 282 |
|  | 120 | 460199 | 552546 | 287 |
|  | 130 | 460209 | 552546 | 292 |
|  | 140 | 460219 | 552547 | 297 |
|  | 150 | 460229 | 552547 | 301 |
|  | 160 | 460239 | 552547 | 307 |
|  | 170 | 460250 | 552548 | 312 |
|  | 180 | 460259 | 552549 | 315 |
|  | 190 | 460269 | 552550 | 319 |
|  | 200 | 460279 | 552552 | 322 |
|  | 210 | 460289 | 552553 | 324 |
|  | 220 | 460299 | 552552 | 328 |
|  | 230 | 460310 | 552549 | 331 |
|  | 240 | 460320 | 552547 | 334 |
|  | 250 | 460329 | 552544 | 337 |
|  | 260 | 460339 | 552548 | 338 |
|  | 270 | 460349 | 552547 | 339 |
|  | 280 | 460359 | 552547 | 339 |
|  | 290 | 460370 | 552547 | 340 |
|  | 300 | 460379 | 552547 | 340 |
|  | 310 | 460389 | 552546 | 341 |
|  | 320 | 460400 | 552546 | 341 |
|  | 330 | 460409 | 552546 | 340 |
|  | 340 | 460419 | 552547 | 338 |
|  | 350 | 460430 | 552548 | 336 |
|  | 360 | 460439 | 552548 | 334 |
|  | 370 | 460449 | 552549 | 333 |
|  | 380 | 460459 | 552550 | 330 |
|  | 390 | 460469 | 552552 | 328 |
|  | 400 | 460479 | 552553 | 326 |

| **Survey Line** | **Distance (m)** | **Easting (m)** | **Northing (m)** | **Elevation (m)** |
| --- | --- | --- | --- | --- |
| CH 16 | 0 | 460080 | 552433 | 229 |
|  | 10 | 460090 | 552435 | 232 |
|  | 20 | 460099 | 552436 | 236 |
|  | 30 | 460110 | 552436 | 240 |
|  | 40 | 460119 | 552437 | 246 |
|  | 50 | 460130 | 552437 | 250 |
|  | 60 | 460139 | 552438 | 256 |
|  | 70 | 460149 | 552437 | 261 |
|  | 80 | 460160 | 552438 | 267 |
|  | 90 | 460169 | 552438 | 271 |
|  | 100 | 460179 | 552439 | 276 |
|  | 110 | 460190 | 552439 | 281 |
|  | 120 | 460199 | 552440 | 286 |
|  | 130 | 460209 | 552440 | 291 |
|  | 140 | 460219 | 552441 | 295 |
|  | 150 | 460229 | 552442 | 300 |
|  | 160 | 460239 | 552442 | 305 |
|  | 170 | 460249 | 552443 | 309 |
|  | 180 | 460259 | 552445 | 313 |
|  | 190 | 460269 | 552447 | 315 |
|  | 200 | 460279 | 552450 | 318 |
|  | 210 | 460288 | 552452 | 320 |
|  | 220 | 460299 | 552452 | 322 |
|  | 230 | 460309 | 552450 | 324 |
|  | 240 | 460319 | 552448 | 326 |
|  | 250 | 460330 | 552446 | 327 |
|  | 260 | 460340 | 552447 | 327 |
|  | 270 | 460349 | 552447 | 327 |
|  | 280 | 460359 | 552446 | 326 |
|  | 290 | 460370 | 552447 | 326 |
|  | 300 | 460379 | 552447 | 325 |
|  | 310 | 460390 | 552447 | 348 |
|  | 320 | 460399 | 552447 | 325 |
|  | 330 | 460409 | 552450 | 324 |
|  | 340 | 460419 | 552450 | 323 |
|  | 350 | 460430 | 552452 | 322 |
|  | 360 | 460439 | 552454 | 321 |
|  | 370 | 460449 | 552454 | 319 |
|  | 380 | 460459 | 552456 | 318 |
|  | 390 | 460468 | 552457 | 317 |
|  | 400 | 460478 | 552458 | 316 |

| **Survey Line** | **Distance (m)** | **Easting (m)** | **Northing (m)** | **Elevation (m)** |
| --- | --- | --- | --- | --- |
| CH 17 | 0 | 460075 | 552331 | 222 |
|  | 10 | 460085 | 552331 | 227 |
|  | 20 | 460095 | 552331 | 230 |
|  | 30 | 460105 | 552331 | 233 |
|  | 40 | 460115 | 552331 | 238 |
|  | 50 | 460125 | 552330 | 243 |
|  | 60 | 460135 | 552329 | 247 |
|  | 70 | 460145 | 552330 | 250 |
|  | 80 | 460155 | 552329 | 253 |
|  | 90 | 460165 | 552330 | 257 |
|  | 100 | 460175 | 552331 | 260 |
|  | 110 | 460185 | 552331 | 263 |
|  | 120 | 460195 | 552330 | 266 |
|  | 130 | 460205 | 552331 | 270 |
|  | 140 | 460215 | 552332 | 273 |
|  | 150 | 460225 | 552333 | 276 |
|  | 160 | 460235 | 552335 | 278 |
|  | 170 | 460245 | 552336 | 281 |
|  | 180 | 460256 | 552338 | 283 |
|  | 190 | 460265 | 552340 | 285 |
|  | 200 | 460274 | 552344 | 286 |
|  | 210 | 460285 | 552346 | 287 |
|  | 220 | 460295 | 552347 | 288 |
|  | 230 | 460305 | 552346 | 289 |
|  | 240 | 460316 | 552345 | 288 |
|  | 250 | 460325 | 552344 | 288 |
|  | 260 | 460335 | 552345 | 288 |
|  | 270 | 460345 | 552346 | 287 |
|  | 280 | 460355 | 552346 | 286 |
|  | 290 | 460365 | 552347 | 285 |
|  | 300 | 460375 | 552348 | 284 |
|  | 310 | 460384 | 552349 | 283 |
|  | 320 | 460395 | 552350 | 282 |
|  | 330 | 460405 | 552351 | 281 |
|  | 340 | 460415 | 552351 | 279 |
|  | 350 | 460425 | 552351 | 278 |
|  | 360 | 460435 | 552351 | 278 |
|  | 370 | 460444 | 552352 | 276 |
|  | 380 | 460454 | 552351 | 275 |
|  | 390 | 460465 | 552352 | 274 |
|  | 400 | 460475 | 552353 | 273 |

| **Survey Line** | **Distance (m)** | **Easting (m)** | **Northing (m)** | **Elevation (m)** |
| --- | --- | --- | --- | --- |
| CH 18 | 0 | 460098 | 552226 | 229 |
|  | 10 | 460112 | 552228 | 231 |
|  | 20 | 460125 | 552230 | 233 |
|  | 30 | 460138 | 552232 | 235 |
|  | 40 | 460150 | 552235 | 237 |
|  | 50 | 460163 | 552236 | 239 |
|  | 60 | 460175 | 552239 | 241 |
|  | 70 | 460184 | 552241 | 243 |
|  | 80 | 460191 | 552242 | 246 |
|  | 90 | 460198 | 552243 | 248 |
|  | 100 | 460207 | 552244 | 248 |
|  | 110 | 460216 | 552246 | 249 |
|  | 120 | 460225 | 552247 | 251 |
|  | 130 | 460237 | 552249 | 253 |
|  | 140 | 460250 | 552251 | 256 |
|  | 150 | 460260 | 552253 | 256 |
|  | 160 | 460271 | 552254 | 257 |
|  | 170 | 460277 | 552256 | 258 |
|  | 180 | 460282 | 552257 | 258 |
|  | 190 | 460288 | 552258 | 258 |
|  | 200 | 460294 | 552259 | 258 |
|  | 210 | 460305 | 552258 | 258 |
|  | 220 | 460315 | 552257 | 258 |
|  | 230 | 460326 | 552256 | 257 |
|  | 240 | 460337 | 552258 | 256 |
|  | 250 | 460346 | 552258 | 255 |
|  | 260 | 460356 | 552258 | 253 |
|  | 270 | 460368 | 552258 | 251 |
|  | 280 | 460377 | 552258 | 250 |
|  | 290 | 460386 | 552258 | 250 |
|  | 300 | 460395 | 552258 | 249 |
|  | 310 | 460407 | 552257 | 248 |
|  | 320 | 460419 | 552258 | 246 |
|  | 330 | 460432 | 552258 | 244 |
|  | 340 | 460442 | 552257 | 243 |
|  | 350 | 460453 | 552257 | 242 |
|  | 360 | 460464 | 552257 | 241 |
|  | 370 | 460473 | 552257 | 241 |
|  | 380 | 460481 | 552257 | 240 |
|  | 390 | 460486 | 552257 | 240 |
|  | 400 | 460492 | 552257 | 240 |

| **Survey Line** | **Distance (m)** | **Easting (m)** | **Northing (m)** | **Elevation (m)** |
| --- | --- | --- | --- | --- |
| CH 19 | 0 | 460085 | 552135 | 205 |
|  | 10 | 460091 | 552135 | 206 |
|  | 20 | 460100 | 552136 | 206 |
|  | 30 | 460110 | 552137 | 207 |
|  | 40 | 460120 | 552139 | 208 |
|  | 50 | 460130 | 552140 | 209 |
|  | 60 | 460140 | 552141 | 210 |
|  | 70 | 460149 | 552141 | 211 |
|  | 80 | 460157 | 552143 | 212 |
|  | 90 | 460165 | 552144 | 213 |
|  | 100 | 460175 | 552147 | 215 |
|  | 110 | 460185 | 552148 | 217 |
|  | 120 | 460193 | 552148 | 219 |
|  | 130 | 460203 | 552149 | 221 |
|  | 140 | 460213 | 552150 | 223 |
|  | 150 | 460223 | 552149 | 225 |
|  | 160 | 460234 | 552150 | 227 |
|  | 170 | 460249 | 552150 | 230 |
|  | 180 | 460263 | 552153 | 231 |
|  | 190 | 460274 | 552154 | 233 |
|  | 200 | 460285 | 552156 | 234 |
|  | 210 | 460295 | 552158 | 236 |
|  | 220 | 460307 | 552156 | 237 |
|  | 230 | 460320 | 552155 | 238 |
|  | 240 | 460333 | 552155 | 238 |
|  | 250 | 460345 | 552153 | 238 |
|  | 260 | 460355 | 552154 | 238 |
|  | 270 | 460365 | 552153 | 237 |
|  | 280 | 460374 | 552153 | 237 |
|  | 290 | 460384 | 552154 | 237 |
|  | 300 | 460392 | 552154 | 237 |
|  | 310 | 460401 | 552154 | 237 |
|  | 320 | 460410 | 552153 | 236 |
|  | 330 | 460418 | 552152 | 236 |
|  | 340 | 460427 | 552152 | 236 |
|  | 350 | 460436 | 552155 | 236 |
|  | 360 | 460444 | 552155 | 236 |
|  | 370 | 460452 | 552155 | 236 |
|  | 380 | 460459 | 552154 | 236 |
|  | 390 | 460467 | 552154 | 236 |
|  | 400 | 460476 | 552155 | 236 |

| **Survey Line** | **Distance (m)** | **Easting (m)** | **Northing (m)** | **Elevation (m)** |
| --- | --- | --- | --- | --- |
| CH 20 | 0 | 460117 | 552031 | 198 |
|  | 10 | 460125 | 552038 | 199 |
|  | 20 | 460130 | 552034 | 200 |
|  | 30 | 460141 | 552038 | 201 |
|  | 40 | 460150 | 552038 | 203 |
|  | 50 | 460159 | 552036 | 204 |
|  | 60 | 460170 | 552037 | 206 |
|  | 70 | 460180 | 552038 | 208 |
|  | 80 | 460189 | 552036 | 209 |
|  | 90 | 460193 | 552045 | 210 |
|  | 100 | 460202 | 552045 | 212 |
|  | 110 | 460210 | 552041 | 216 |
|  | 120 | 460217 | 552047 | 216 |
|  | 130 | 460228 | 552044 | 220 |
|  | 140 | 460236 | 552047 | 222 |
|  | 150 | 460244 | 552050 | 223 |
|  | 160 | 460253 | 552046 | 226 |
|  | 170 | 460265 | 552046 | 227 |
|  | 180 | 460271 | 552050 | 228 |
|  | 190 | 460282 | 552049 | 229 |
|  | 200 | 460293 | 552052 | 231 |
|  | 210 | 460306 | 552052 | 233 |
|  | 220 | 460313 | 552053 | 233 |
|  | 230 | 460322 | 552054 | 235 |
|  | 240 | 460333 | 552052 | 236 |
|  | 250 | 460348 | 552052 | 237 |
|  | 260 | 460360 | 552056 | 237 |
|  | 270 | 460377 | 552051 | 238 |
|  | 280 | 460389 | 552059 | 238 |
|  | 290 | 460398 | 552061 | 238 |
|  | 300 | 460410 | 552059 | 239 |
|  | 310 | 460422 | 552059 | 239 |
|  | 320 | 460432 | 552059 | 239 |
|  | 330 | 460437 | 552056 | 240 |
|  | 340 | 460444 | 552059 | 240 |
|  | 350 | 460453 | 552052 | 240 |
|  | 360 | 460457 | 552056 | 240 |
|  | 370 | 460466 | 552058 | 240 |
|  | 380 | 460475 | 552061 | 240 |
|  | 390 | 460479 | 552058 | 241 |
|  | 400 | 460487 | 552058 | 241 |

| **Survey Line** | **Distance (m)** | **Easting (m)** | **Northing (m)** | **Elevation (m)** |
| --- | --- | --- | --- | --- |
| CH 21 | 0 | 460095 | 551937 | 208 |
|  | 10 | 460111 | 551939 | 212 |
|  | 20 | 460128 | 551937 | 216 |
|  | 30 | 460146 | 551932 | 217 |
|  | 40 | 460160 | 551941 | 219 |
|  | 50 | 460175 | 551943 | 222 |
|  | 60 | 460188 | 551943 | 224 |
|  | 70 | 460197 | 551940 | 226 |
|  | 80 | 460203 | 551941 | 226 |
|  | 90 | 460209 | 551947 | 227 |
|  | 100 | 460216 | 551948 | 228 |
|  | 110 | 460222 | 551947 | 230 |
|  | 120 | 460230 | 551940 | 230 |
|  | 130 | 460239 | 551949 | 231 |
|  | 140 | 460246 | 551954 | 232 |
|  | 150 | 460253 | 551948 | 233 |
|  | 160 | 460261 | 551949 | 234 |
|  | 170 | 460269 | 551954 | 235 |
|  | 180 | 460278 | 551953 | 236 |
|  | 190 | 460286 | 551953 | 236 |
|  | 200 | 460312 | 551954 | 237 |
|  | 210 | 460305 | 551954 | 238 |
|  | 220 | 460322 | 551953 | 239 |
|  | 230 | 460327 | 551954 | 240 |
|  | 240 | 460337 | 551957 | 240 |
|  | 250 | 460347 | 551953 | 241 |
|  | 260 | 460353 | 551953 | 241 |
|  | 270 | 460364 | 551953 | 241 |
|  | 280 | 460377 | 551950 | 242 |
|  | 290 | 460387 | 551949 | 242 |
|  | 300 | 460394 | 551950 | 242 |
|  | 310 | 460402 | 551951 | 243 |
|  | 320 | 460409 | 551951 | 243 |
|  | 330 | 460415 | 551947 | 243 |
|  | 340 | 460427 | 551949 | 244 |
|  | 350 | 460435 | 551944 | 245 |
|  | 360 | 460443 | 551945 | 245 |
|  | 370 | 460451 | 551951 | 245 |
|  | 380 | 460458 | 551948 | 245 |
|  | 390 | 460466 | 551949 | 245 |
|  | 400 | 460473 | 551952 | 245 |
